# Prior Acoustic Trauma Alters Type II Afferent Activity in the Mouse Cochlea

**DOI:** 10.1523/ENEURO.0383-21.2021

**Published:** 2021-11-02

**Authors:** Nathaniel Nowak, Megan Beers Wood, Elisabeth Glowatzki, Paul Albert Fuchs

**Affiliations:** 1 Department of Otolaryngology, Head and Neck Surgery, Johns Hopkins University School of Medicine, Baltimore, MD 21205; 2 Department of Neuroscience, Johns Hopkins University School of Medicine, Baltimore, MD 21205

**Keywords:** acoustic trauma, ATP, auditory neuroscience, glia, nociception, peripheral nervous system

## Abstract

Auditory stimuli travel from the cochlea to the brainstem through type I and type II cochlear afferents. While type I afferents convey information about the frequency, intensity, and timing of sounds, the role of type II afferents remains unresolved. Limited recordings of type II afferents from cochlear apex of prehearing rats reveal they are activated by widespread outer hair cell stimulation, ATP, and by the rupture of nearby outer hair cells. Altogether, these lines of evidence suggest that type II afferents sense loud, potentially damaging levels of sound. To explore this hypothesis further, calcium imaging was used to determine the impact of acoustic trauma on the activity of type II cochlear afferents of young adult mice of both sexes. Two known marker genes (*Th*, *Drd2*) and one new marker gene (*Tac1*), expressed in type II afferents and some other cochlear cell types, drove GCaMP6f expression to reveal calcium transients in response to focal damage in the organ of Corti in all turns of the cochlea. Mature type II afferents responded to acute photoablation damage less often but at greater length compared with prehearing neurons. In addition, days after acoustic trauma, acute photoablation triggered a novel response pattern in type II afferents and surrounding epithelial cells, delayed bursts of activity occurring minutes after the initial response subsided. Overall, calcium imaging can report type II afferent responses to damage even in mature and noise-exposed animals and reveals previously unknown tissue hyperactivity subsequent to acoustic trauma.

## Significance Statement

The function of type II cochlear afferents is currently unknown. The prevailing hypothesis is that these neurons detect excessively loud sound and tissue damage within the cochlea. However, this hypothesis has not been directly investigated in fully hearing, mature animals. To this end, we show that type II afferents in mature mice experience prolonged calcium transients in response to focal tissue damage as compared with young, prehearing mice. Previous traumatic noise exposure caused novel delayed response patterns. Together, our data support the role of type II cochlear afferents as tissue damage detectors in the cochlea and suggest that changes in type II afferent activity may contribute to pathologies resulting from traumatic noise exposure.

## Introduction

**Figure 1.**
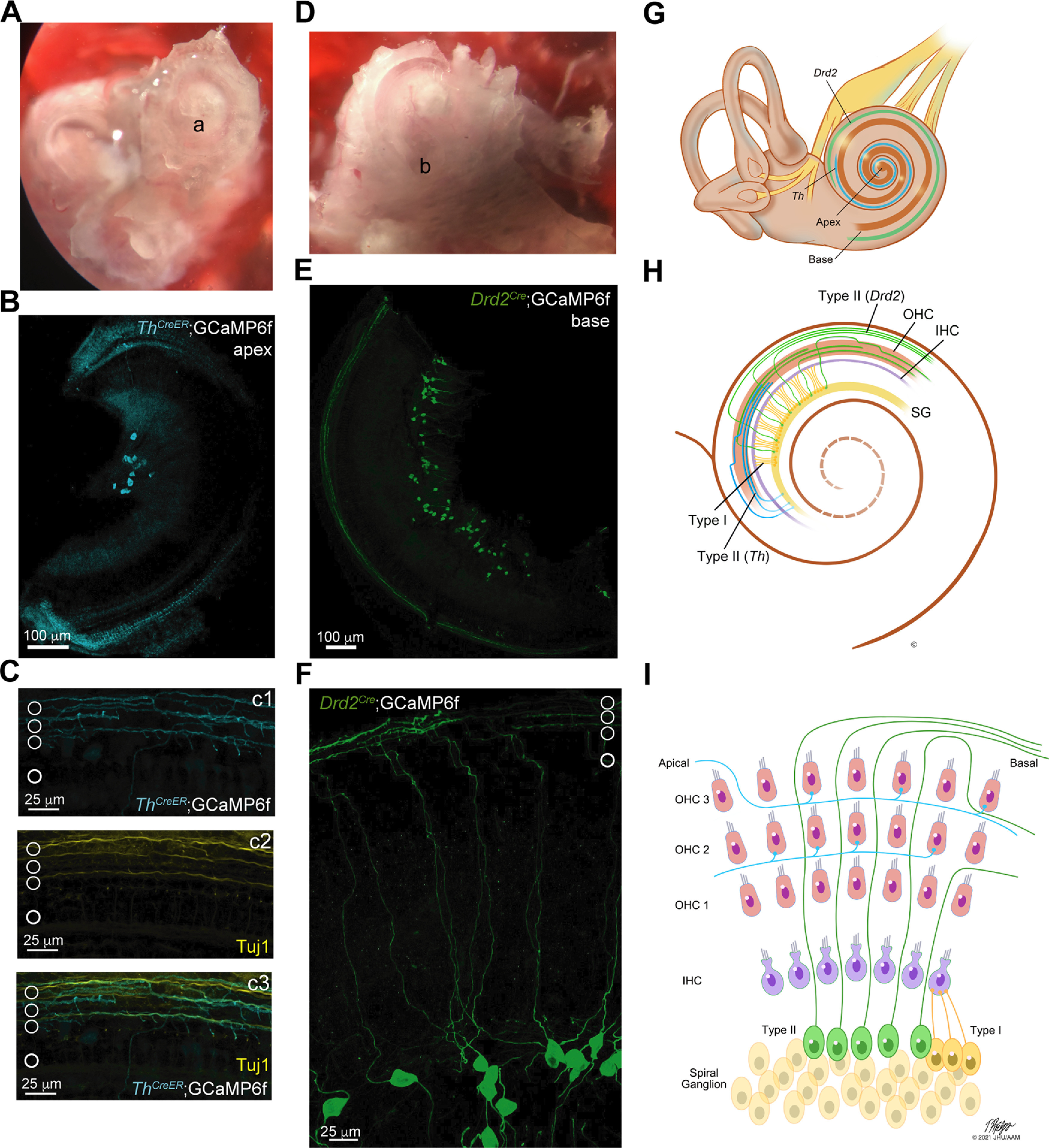
Type II afferent-associated genes drive GCaMP6f expression. ***A***, Image taken through a dissecting microscope of an excised, otic capsule preparation with access to the apical turn; “a” indicates the location of the tip of the apical turn of the cochlear epithelium. ***B***, Maximum intensity projection image of a whole mount preparation of the apical turn of a *Th^2A-CreER^*;GCaMP6f mouse. The GFP expression associated with GCaMP6f expression has been pseudo-colored cyan. ***C***, Maximum intensity projection image of *Th^2A-CreER^*;GCaMP6f expression in the peripheral dendrites of apical type II afferent neurons. GCaMP6F expressing type II afferents are labeled in cyan (***c1***), the pan-neuronal marker Tuj1 is labeled in yellow (***c2***). The overlay with Tuj1 (***c3***, green) shows *Th^2A-CreER^*;GCaMP6f expression in neurons in the OHC region of the organ of Corti as indicated by the white circles. ***D***, Image taken through a dissecting microscope of an excised, otic capsule preparation with access to the basal turn; “b” indicated the end of the basal turn of the cochlea. ***E***, Maximum intensity projection image of a whole mount preparation of the basal turn of a *Drd2^Cre^*;GCaMP6f mouse. The GFP expression associated with GCaMP6f expression is shown in green. ***F***, Maximum intensity projection image of *Drd2^Cre^*;GCaMP6f expression in the somata and peripheral dendrites of basal type II afferent neurons. Two 40× magnification images were stitched using the pairwise stitching plugin in ImageJ to visualize the path of the neuron from soma to organ of Corti. Hair cell location is indicated by white circles. ***G***, Diagram showing the tonotopic expression patterns of *Th* (cyan) and *Drd2* (green) in the cochlea. ***H***, Diagram showing the morphology of type II afferent neurons in the middle turn of the cochlea. Type II morphology includes a dendritic arbor with a characteristic 90° turn toward the base of the cochlea. *Th* (cyan); *Drd2* (green). ***I***, Diagram of the morphologic differences between type I afferent neurons (yellow) and type II afferent neurons (*Drd2-*expressing, green; *Th*-expressing, cyan) in the organ of Corti.

The spiral ganglion neurons (SGNs) of the cochlea are divided into type I afferents that sensitively encode sound intensity, timing, and frequency, and type II afferents that respond only to loud, broadband sound (Robertson, 1984; Brown, 1994; Robertson et al., 1999; Flores et al., 2015; Weisz et al., 2021). Type I afferents constitute 90–95% of all SGNs, with type II afferents making up the remaining 5% (Perkins and Morest, 1975; Berglund and Ryugo, 1987). The two types of SGNs differ not only in proportion but also in innervation pattern as illustrated in Figure 1*H*,*I*. Each myelinated type I afferent synapses with one inner hair cell (IHC) and each IHC is contacted by 10–30 unbranched type I afferent dendrites (Liberman, 1982). The small, unmyelinated type II afferents make a 90° turn at the level of the outer hair cells (OHCs) and then extend a long dendritic arbor toward the cochlear base, contacting an average of 24 OHCs (range 9–100; Smith, 1975; Kiang et al., 1982; Berglund and Ryugo, 1987; Weisz et al., 2012; Martinez-Monedero et al., 2016; Ghimire and Deans, 2019).

It has been difficult to determine the function of type II afferents. *In vivo* electrophysiological recordings are rare because of the scarcity and small caliber of type II axons (Brown, 1994; Robertson et al., 1999). Of the limited recordings of putative type II afferents within the eighth nerve, only one had a response to loud, broadband sound (Robertson, 1984; Brown, 1994). In a mouse model where type I signaling from IHCs was interrupted, type II afferents were shown to respond only to excessively loud sound (Flores et al., 2015; although see Weisz et al., 2021). Intracellular recording in apical cochlear segments from prehearing animals showed that type II afferents respond weakly to glutamate release from OHCs but strongly to exogenous ATP (Weisz et al., 2009, 2012). The rupture of OHCs activated type II afferents in prehearing animals via connexin-dependent ATP release (Liu et al., 2015), presumably from supporting cells (Gale et al., 2004; Lahne and Gale, 2008, 2010). The emerging hypothesis is that acoustic trauma activates type II cochlear afferents directly and through interactions with surrounding epithelia, by analogy with cutaneous nociception (Talagas et al., 2020).

Thus, type II afferents may function as auditory nociceptors. However, the stimuli that drive type II afferents in mature, hearing animals have not been elucidated. In addition, previous cellular studies have been limited to tissues excised from the more accessible apical turn of the cochlea. Damage in the mature cochlea, such as OHC loss because of acoustic trauma, can cause ATP- mediated calcium waves propagated through supporting cells (Gale et al., 2004; Anselmi et al., 2008; Sirko et al., 2019) similar to those occurring within Kölliker’s organ during development (Tritsch et al., 2007, 2010). The present work presents calcium imaging of type II afferent responses to acute trauma in all turns of the prehearing and mature cochlea. In all conditions, type II afferents retain the ability to respond to focal ablation but less reliably in mature cochleas. Additionally, a new mouse model links this activation of type II afferents to calcium waves in epithelial cells. Significantly, the responses of type II afferents and surrounding epithelia to focal ablation are enhanced after noise-induced hearing loss.

## Materials and Methods

### Mice

Five mouse models were used in these studies (Table 1). *Th^2A-CreER^* (Abraira et al., 2017) and *Drd2^Cre^* homozygous mouse lines were backcrossed to the C57BL/6J strain and maintained independently. F1 offspring of either sex of *Th^2A-CreER^*, *Drd2^Cre^*, or *Tac1^Cre^* bred with homozygous GCaMP6f^fl/fl^ mice were generated for the experiments in this paper (Table 1). Tamoxifen (Sigma-Aldrich, catalog #T5648) was administered by gavage at P3–P4 for the *Th^2A-CreER^*;GCaMP6f animals at a dosage of 0.2 mg in corn oil (Sigma-Aldrich, catalog #C8267). Prehearing animals were defined as postnatal 6–10 d of age (Liu et al., 2015). Mature mice were defined as adult mice >6 and <10 weeks of age to avoid any confounding effects of accelerated age-related hearing loss associated with the C57BL/6J strain (Johnson et al., 1997, 2017). Care of the animals followed all institutional guidelines of the Animal Care and Use Committee of Johns Hopkins University School of Medicine.

**Table 1:** Mouse genotypes and sources

Name	Genotype	Source
*Th^2A-CreER^*		Abraira et al. (2017)
*Drd2^Cre^*	B6.FVB(Cg)-Tg(Drd2-cre)ER44Gsat/Mmucd	RRID:MMRRC_032108-UCD
*Tac1^Cre^*	B6;129S-*Tac1^tm1.1(cre)Hze^*/J	IMSR catalog #JAX:021877, RRID:IMSR_JAX:021877
GCAMP6f*^fl^*^/^*^fl^*	C57BL/6N-*Gt(ROSA)26Sor^tm1(CAG-GCaMP6f)^Khakh* /J	IMSR catalog #JAX:029626, RRID:IMSR_JAX:029626
Ai9	B6.Cg-*Gt(ROSA)26Sor^tm9(CAG-tdTomato)Hze^*/J	IMSR catalog #JAX:007909, RRID:IMSR_JAX:007909

Leftmost column provides the name as used in the text for each mouse line. The left column shows the full mouse line name as found on The Jackson Laboratory website. The right column shows the source of each of the mouse lines.

### Calcium imaging

Mice were euthanized based on American Veterinary Medical Association (AVMA) guidelines appropriately for the age of the animal immediately before imaging. Prehearing animals were placed on a heating pad to maintain core body temperature before euthanasia. The otic capsule preparation was adapted from the *in situ* cochlea preparation described in Sirko and colleagues, wherein the hemidissected head was placed in 2.5 mm K^+^ external solution on ice and the otic capsule excised from the temporal bone (Sirko et al., 2019). Then, the bone overlying the cochlear epithelium was removed. The fenestrated otic capsule cochlea was mounted on utility wax for observation at room temperature. A gravity perfusion system was constructed on a motorized stage of a 710 LSM Zeiss microscope with a GaAsP detector (Zeiss) and Chameleon two-photon laser (Coherent). The preparation was placed in external solution and imaged using a 20× non-coverslip-corrected water-immersion objective (Nikon). With an objective in place, the two-photon laser measured 299 mW at 100% power when set to the 920-nm wavelength used in these experiments.

### Solutions for bath application

2.5 mm K^+^ external solution was composed of 2.5 mm KCl, 148 mm NaCl, 1.3 mm CaCl_2_, 0.9 mm MgCl_2_, 0.7 mm NaH_2_PO_4_, 5.6 mm D-glucose, and 10 mm HEPES (∼300 mOsm; pH 7.4; NaOH); 40 mm K^+^ external solution was composed of 40 mm KCl, 104 mm NaCl, 1.3 mm CaCl_2_, 0.9 mm MgCl_2_, 0.7 mm NaH_2_PO_4_, 5.6 mm D-glucose, and 10 mm HEPES (∼300 mOsm; pH 7.4; NaOH). (All components of the external solution came from Sigma-Aldrich.) Alternative concentrations of K^+^ were made by diluting the 40 mm K^+^ external solution with proportions of 2.5 mm K^+^ external solution to achieve the desired concentration. For experiments testing the effect of P2X receptor blockade, pyridoxalphosphate-6-azophenyl-2',4'-disulfonic acid (PPADS; Tocris Biosciences, catalog # 0683) was added to the 2.5 mm K^+^ extracellular solution to achieve a 100 μm concentration.

### Ca^2+^ signaling image acquisition

Zen Black software was used to control the Chameleon two-photon laser. Imaging parameters were set at 2× digital zoom and 512 × 128 pixels allowing for acquisition of time series at 80 ms a frame. Imaging was performed at 8% laser power. Type II afferents were identified in the presence of 15 or 40 mm K^+^ external solution then the solution was replaced with 5.8 mm K^+^ external solution before assessing calcium fluorescence changes. For laser ablation, laser power was increased to 100% with up to 1000 iterations within a hand-drawn region of interest (ROI). Because of the sensitivity of the GaAsP detector, the sample was not imaged during laser ablation for a median time of 8.4 s.

Occasionally, multiple videos from the same animal were taken over the course of one experimental session. Videos were not taken in the same location after the tissue was damaged. Instead, videos were taken at a location with healthy tissue tens of microns apical to the site of damage to avoid recording from the same neurons.

### Quantification of change in fluorescence

Time series images were transformed into a projection image using SD for the projection type In ImageJ. This type of projection image highlighted the areas of the field of view with the largest changes in fluorescence within a black and white 255-point scale. ROIs were created using the polygon selection tool with care to prevent overlapping regions. One region was selected as the background; the values from this region were subtracted from each of the other regions (ΔF). The equivalent of 30 s from the control condition of the time series were used as the baseline fluorescence of all regions (F_baseline_). The resulting equation is thus:

$$\Delta \text{F}/\text{F }=\;({\text{F}}_{\text{ROI}}-{\text{ F}}_{\text{background}})/{\text{F}}_{\text{baseline.}}$$

The data were then plotted as ΔF/F for every ROI in each frame. All results are represented as fold-change of ΔF/F with 1 representing baseline and 2 representing a doubling of calcium fluorescence. An ROI was considered responsive if its max ΔF/F was >3 SDs above the baseline before laser ablation and/or at the end of the recording. Neuronal area was calculated by dividing the total area of the ROIs containing a responsive neuronal segment divided by the total area of all neuronal ROIs visible in frame.

To compare between videos and genotypes, traces were scaled with the maximum set at 1 (ΔF/F = 1). This allowed for calculations of response duration within responsive ROIs. Total response duration includes the time of the laser ablation to the time at which the trace has fallen to within 3 SDs of the baseline fluorescence for at least five consecutive frames. Time constants of decay (tau) were estimated by fitting to exponential decays using the Excel add-in Solver.

### Non-GCaMP6f-associated fluorescence (NGAF) magnitude analysis

Videos were analyzed in ImageJ. All pixels above a brightness level of 30 on a 0–255 scale were counted for each frame of the video (Fig. 5, raw trace). A sliding average of the previous 30 s was subtracted to generate the plots in Figures 5*D*,*H*, 9*C*. The area under the curve measurements were derived using the Trapz function of MATLAB. The area under the curve of all positive deflections (arbitrary units, a.u.) describes the magnitude of the NGAF response.

### Immunofluorescence

Whole-mount immunostaining and imaging was performed as previously described (Vyas et al., 2019). Briefly, grossly dissected inner ears were perfused with 4% paraformaldehyde (PFA; Electron Microscopy Sciences) in 1× PBS (Quality Biological Inc.) through the round window, then postfixed for 30 min on a 3D-Rotator. Mature inner ears were washed (3 × 30 min) in PBS, then decalcified in 125 mm EDTA (Quality Biological Inc.) in PBS for 2 h before dissection into turns of the organ of Corti. Postnatal day 7–10 (P7–10) inner ears were not decalcified before washing in PBS (3 × 30 min) and dissection into turns of the organ of Corti. Cochlear turns were immunolabeled with Goat anti-GFP (Sicgen, catalog #A32814, RRID:AB_2333099) to label GCaMP6f protein. Donkey anti-goat Alexa Fluor 488 (Invitrogen, catalog #AB0020-200, RRID:AB_2762838) was used along with Alexa Fluor 647-conjugated phalloidin (Thermofisher Scientific, catalog #A22287, RRID:AB_2620155) and 4'6-diamidine-2'-phenylindole dihydrochloride (DAPI; Roche Molecular Systems) for GCaMP6f, hair cells, and nuclei, respectively (Fig. 1). Cochlear turns were mounted using ProLong Fade Gold Antifade Mountant (Thermofisher Scientific, catalog #P36930) and imaged on a 700 LSM Zeiss confocal microscope.

### Immunofluorescence for *Tac1^Cre^*;Ai9 animals

Immunofluorescence was performed as described in the main methods sections with the following differences. Dorsal root ganglia were dissected from mice as previously described (Sleigh et al., 2016). Dorsal root ganglia were not decalcified. Cochleas and dorsal root ganglia from *Tac1^Cre^*; Ai9 animals were immunolabeled with the following polyclonal antibodies: goat anti-tdTomato (Sicgen, catalog #AB8181-200, RRID:AB_2722750) and rabbit anti-CGRP-α (Immunostar, Hudson, WI, catalog # 24112, RRID:AB_572217). Alexa Fluor 568-conjugated donkey anti-goat (Invitrogen, catalog #A-11057, RRID:AB_142581) and Alexa Fluor 488-conjugated donkey anti-rabbit (Invitrogen, catalog #A-21206, RRID:AB_141708) secondary antibodies were used for tdTomato and CGRP-α, respectively. *Tac1^Cre^*; Ai9 quantification of SGN neurons was based on the previously published procedure for counting SGNs (Wu et al., 2018). Briefly, the organ of Corti was divided into bins of 10% of the total length beginning at the apex (0%) to the base (100%) and straight tangential lines segmented the SGN. SGNs within each bin were counted.

### Auditory brainstem response (ABR)

The ABR system, procedures and quantification software used for this study have been previously described (Lauer and May, 2011; Lina and Lauer, 2013). Mice were anesthetized with an intraperitoneal injection of 0.1 ml per 20 g body weight of a mixture of ketamine (100 mg/kg) and xylazine (20 mg/kg) in 14% ethanol before being placed on a gauze covered heating pad in the ABR chamber. The animals’ eyes were swabbed with petrolatum-based ophthalmic ointment to prevent corneal ulcers during anesthesia. Subdermal platinum electrodes were placed at the vertex of the head (non-inverting), the left pinna (inverting), and on the left side at the base of the tail (ground). A total of 300 repetitions of a click or pure-tone stimulus (10 stimuli/s) were used to generate averaged ABR waveforms. Each tonal stimulus was 5 ms in duration with a 0.5-ms rise and fall time. A Fostex dome tweeter speaker (model FT28D) in a foam-lined chamber was used to present the stimuli to mice 30 cm away. The ABR threshold was defined with custom MATLAB software by calculating the averaged peak-to-peak voltage during a 5-ms interval, beginning 1 ms after the onset of the stimulus, compared with the averaged peak-to-peak voltage in a 5-ms window 20 ms after the stimulus. The threshold was determined as the stimulus level where the peak-to-peak response was >2 SDs above the electrical noise.

### Noise exposure(s)

Mice were transferred to a low-noise, satellite housing facility from the day before noise exposure through the endpoint for calcium imaging experiments. Because of the susceptibility of the background strain, C57BL/6J, to age-related hearing loss (Johnson, et al., 1997), all experiments were performed with six-week-old mice. Awake, unrestrained mice were exposed to 110 dB SPL white noise for 2 h in a set of interconnected cages fabricated from wire mesh. Two set-ups were used to perform noise exposure. The first sound chamber and noise exposure set-up has been previously described (NE-1; Wu, et al., 2020). All other animals were exposed to noise in a sound attenuating chamber with a reverberant lining (NE-2; 58 × 40 × 30 cm; width, depth, height). NE-2 was equipped with three overhead, dome tweeter speakers (Promaster TW47 1200W). Speakers were ∼25 cm above the heads of the mice. Broadband noise was generated by 2 JKT tone and noise generators (KV2 audio, Milevsko) powered by Neewer nw-100 phantom power sources (Shenzhen Neewer Technology). The noise generators were connected to two Crown Drivecore XLS2502 amplifiers (Harman): one driving a central speaker in bridge mode and the other driving the two peripheral speakers in input Y mode. The decibel level was tested in each set-up using a Larson Davis LXT sound level meter (PCB Piezotronics) with a 1/2-in free field microphone. Care was taken to measure the sound level at the position of the head of the experimental animals. The spectrum of the noise stimulus was broadband with the highest energy from 2 to 20 kHz as measured by the Larson Davis LXT sound level meter.

### Experimental design and statistical analysis

Power analysis was not performed to determine sample size. Instead, experimental groups were designed to have more than seven mice per group as has been used for similar experiments (Weisz et al., 2009; Liu et al., 2015). Proportionate numbers of both sexes were used throughout all experiments. Data were processed and analyzed in R studio, GraphPad Prism, and Excel. The proportion of videos where at least one neuronal ROI responds to focal ablation (neuronal response rate) is defined by the number of videos with at least one responsive neuronal ROI divided by the total number of videos with at least one visible, neuronal ROI responsive to 40 mm K^+^ external solution. The proportion of videos with visible NGAF over all videos is defined as the NGAF response rate. Statistical testing for neuronal and NGAF response rate data used a generalized linear model with the family set to binomial. Paired data within the same animal used the Wilcoxon ranked-sum test. Data for ABR results and SGN counting were analyzed with ANOVA and used Dunnet’s comparison *post hoc* test for multiple comparisons for the data in Figure 6*E*. Data that stemmed from uneven numbers of multiple recordings across mice were analyzed with a linear mixed model with the mouse identity as the random effect. Fixed effects are listed before each *p* value in Results. For fixed effects or other tests with more than two levels a Bonferroni correction was used to account for multiple comparisons. In figures, asterisks are used to represent *p* values as follows: *p < 0.05, **p < 0.01, ***p < 0.001, ****p < 0.0001. Correlation statistics were calculated with a linear regression. Original data and R files for statistical testing are available on request.

## Results

### Type II afferent-associated genes drive GCaMP6f expression

Type II afferents express several genes that differentiate them from type I afferents. Thus, floxed fluorescent reporters driven by Cre-recombinase under the promoters of these genes permit selective targeting of type II afferents. Two distinctive genes include tyrosine hydroxylase (*Th*), which encodes the rate-limiting enzyme for the production of dopamine, and *Drd2,* which encodes a dopamine receptor subunit (Molinoff and Axelrod, 1971; Kebabian and Calne, 1979; Daubner et al., 2011). *Th^2A-CreER^*;GCaMP6f provided an apically-biased gradient of type II cochlear afferent expression and *Drd2^Cr^*^e^;GCaMP6f targeted basally-biased type II cochlear afferents as schematized in Figure 1*H*. Expression of GCaMP6f was confirmed first with immunohistochemistry and recapitulated the reported expression patterns for each genotype (Fig. 1*B*,*E*; Vyas et al., 2017, 2019; Wu et al., 2018). Cre-driven expression of floxed GCaMP6f was especially apparent in the peripheral dendrites of type II afferents underneath the rows of OHCs for both genotypes (Fig. 1*C*,*F*) even more so than Tuj1 immunostaining, a general neuronal marker (Fig. 1*c1–c3*). Medial olivocochlear neurons as confirmed by Tuj1 staining showed no appreciable expression of GCaMP6f; but, there was some expression in lateral olivocochlear neurons (LOCs) in *Th^2A-CreER^*;GCaMP6f and *Drd2^Cre^*;GCaMP6f animals as described previously for tdTomato expression (Fig. 1*C*; Vyas et al., 2019; Wu et al., 2020). However, LOC neurites are restricted to the IHC region and thus never interfered with imaging type II afferents under the OHCs.

Two-photon microscopy was used to image type II afferents in an otic capsule preparation (Sirko et al., 2019; Fig. 1*A*,*D*,*G*). This allowed for imaging of type II afferents throughout the cochlear spiral. For animals over four weeks of age, the spiral otic capsule was excised from the temporal bone. The apical cochlear turn was revealed by removing the bony tip of the cochlea (Fig. 1*A*). Further dissection revealed more basal regions (Fig. 1*D*). This method preserved the architecture of the older cochlear tissue in contrast to complete soft tissue excision as used in previous studies. Additionally, the otic capsule preparation reduced the amount of damage to the tissue, especially in older cochleas when the bone has ossified. In prehearing animals, both the acutely excised preparation and otic capsule preparation were used, but the otic capsule preparation was preferred to mirror the conditions of the mature preparations. While the otic capsule preparation is quicker and causes less tissue disruption, the tissue is uneven and much thicker, requiring multiphoton microscopy to overcome the curvature and opacity of the tissue.

### Description of calcium imaging protocol

**Figure 2.**
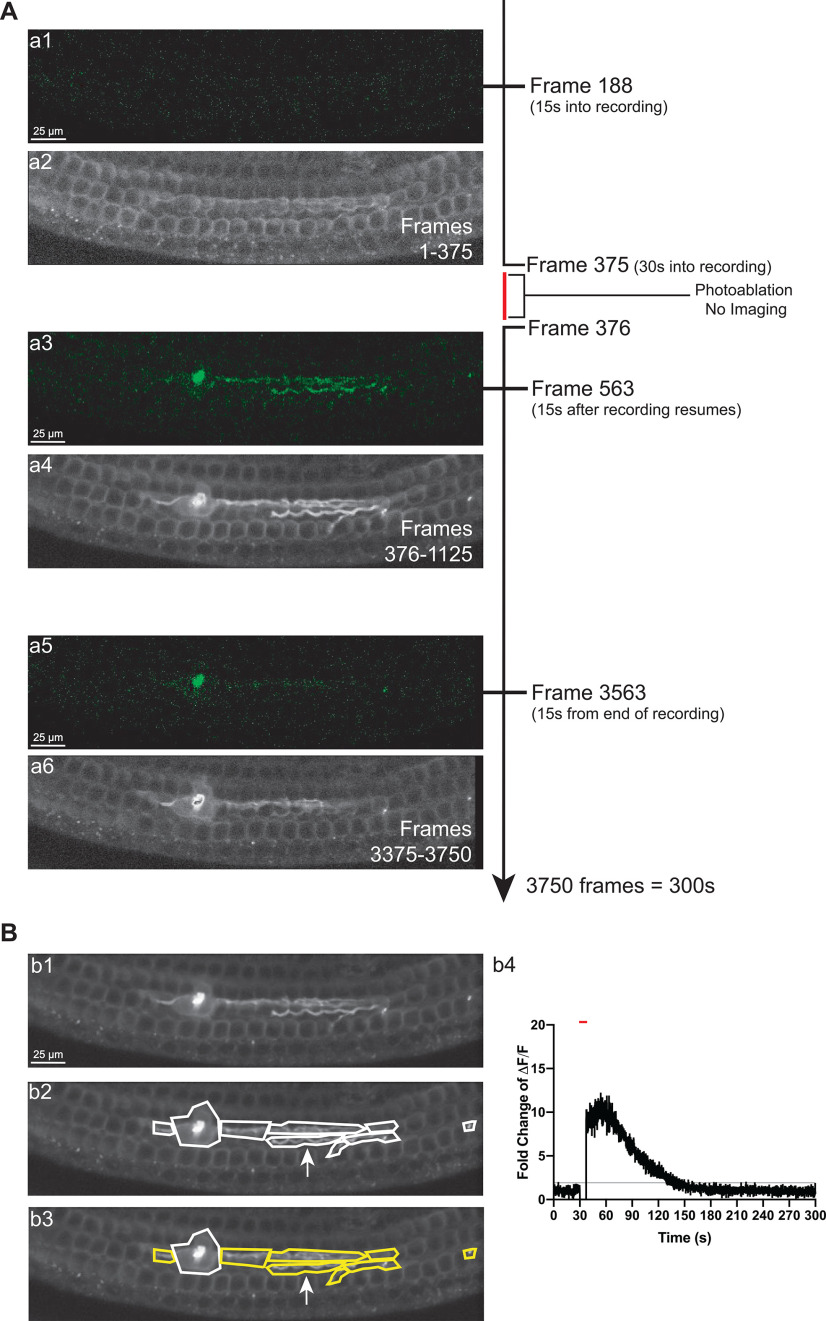
Experimental design for testing type II afferent response to acute damage. ***A***, Diagram of typical photoablation experiment of a *Th^2A-CreER^*;GCaMP6f animal with three time points illustrated. A timeline of the experimental recording is to the right (not to scale). The timeline is broken after video frame 375 (30 s into the recording) to indicate the time when the laser is active, and no imaging occurred. The imaging begins again at frame 376. ***a1***, ***a2***, Image still of raw footage from frame 188 (15 s into the recording) and an SD projection image of the first 375 frames corresponding to the first 30 s of the recording before photoablation. ***a3***, ***a4***, Image still of raw footage from frame 563 (15 s after photoablation ends), and an SD projection image of frames 376–1125 corresponding to the 30 s following the photoablation ending. ***a5***, Still of raw footage from frame 3563 (15 s before the end of the recording). ***a6***, An SD projection image of frames 3375–3750 corresponding to the last 30 s of the recording. ***B***, SD image of the all frames from the recording shown in ***A*** (***b1***), the same image with hand-drawn ROIs around neurons and the site of damage (***b2***). Arrow points to ΔF/F traces of a ROI (***b4***). Gray portion of the graph represents the area between three SDs of the first 375 frames and last 375 frames of the trace. The trace’s peak rises above and returns to a steady state below the gray area. This criterion marks the trace from the ROI as responsive. Responsive ROIs are outlined with yellow (***b3***). Red lines above graphs indicate when the laser is on; since no imaging is occurring the trace is set to 0 at this time. Scale bars: 25 μm.

GCaMP6f signals were recorded from type II afferents in prehearing tissue following focal ablation of nearby OHCs. Experiments followed a stereotyped protocol illustrated in Figure 2. First, type II afferent dendrites were visualized underneath the rows of OHCs in the presence of 15 or 40 mm K^+^ solution where fluorescence in the dendrites was consistently bright. Once dendrites were located, 5.8 or 2.5 mm K^+^ solution replaced the bath solution to recover possible desensitization from the high potassium solution and establish a baseline fluorescence level. Type II afferent activity was recorded for 30 s at the start of each video to establish the baseline for normalization of calcium responses (Fig. 2*a1*,*a2*). OHCs were ruptured through photoablations caused by iterations of 100% power from a two-photon laser focused on a hand-drawn ROI the size of one to three OHCs (Gale et al., 2004; Sirko et al., 2019). Imaging could not occur during laser ablation, therefore a gap occurs in imaging in each trial for a median time of 8.4 s represented by a red bar in the timeline (Fig. 2*A*) and above recordings (Fig. 2*b4*). After this time, imaging typically lasted for ∼4.5 min after the end of laser ablation to capture the full response of the type II afferents (Fig. 2*a3–a6*,*b4*). Dendrites were visualized by generating projection images where each pixel was scaled in gray according to its change in brightness relative to the SD of the baseline fluorescence over either part of or for the entire recording (SD image; Fig. 2*a2*,*a4*,*a6*,*b1–b3*). Once type II dendrites were located in SD images, hand-drawn ROIs were drawn in ImageJ around each visible portion of the neuron, making sure to avoid overlap between multiple dendrites (Fig. 2*b2*). The fluorescence levels for each neuronal ROI were measured for each frame. Then the fluorescence of a background ROI was subtracted from the neuronal fluorescence for each frame. The difference of these values was then divided by the average fluorescence of the neuronal ROI in the 30 s baseline period (ΔF/F). The ΔF/F values for each ROI signify the fold change in brightness over the baseline with 1 representing baseline brightness and a value of 2 as a doubling in the brightness. Each individual ROI was classified as responsive if its ΔF/F trace rose above 3 SDs from the baseline brightness and decreased by at least 3 SDs before reaching the eventual steady state value (Fig. 2*b4*). Individual ROIs that met this criterion are shown as yellow outlined ROIs in each figure. The white arrow in Figure 2*b2*,*b3* indicates the trace for an example responding ROI.

### Tissue damage can evoke calcium responses in apical and basal type II cochlear afferents

**Figure 3.**
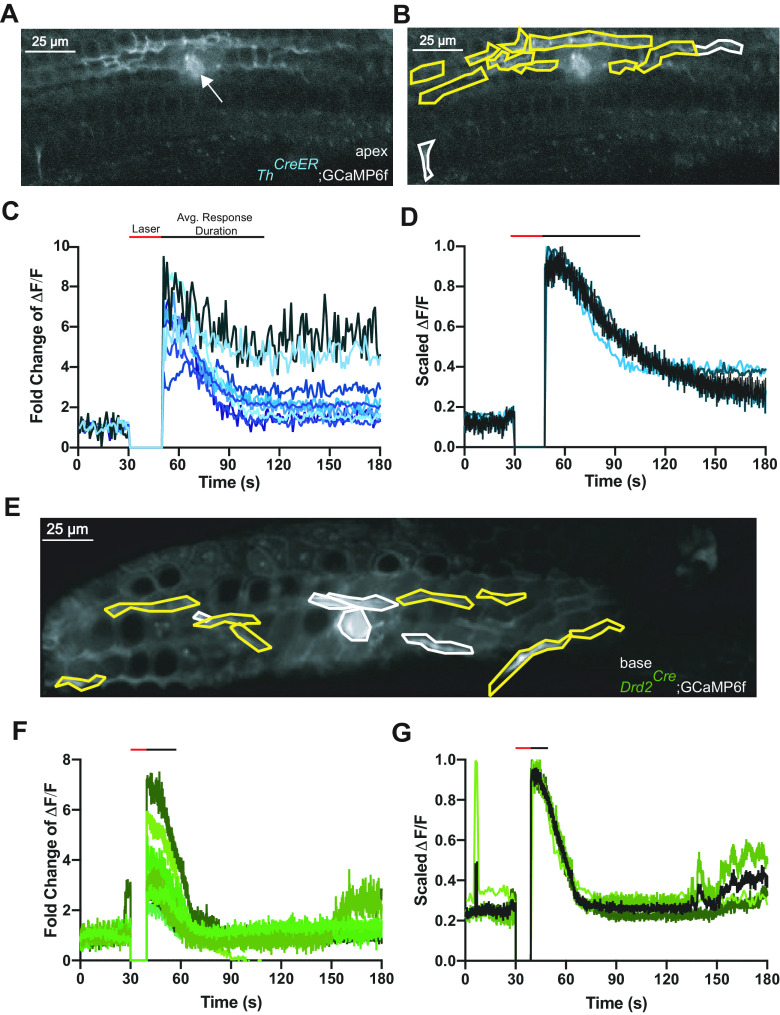
Focal photoablation causes transient calcium events in apical and basal prehearing type II cochlear afferents expressing GCaMP6f. ***A***, An SD image of a prehearing *Th^2A-CreER^*;GCaMP6f mouse cochlea after focal ablation of OHCs with 1000 iterations of 100% laser power. Arrow points to location of laser ablation. ***B***, Same image as ***A*** but with hand drawn ROIs overlaid. Responsive ROIs are outlined in yellow. Scale bars: 25 μm. ***C***, ΔF/F traces of responsive ROIs from ***B***. ΔF/F at time of photoablation and until imaging restarts is set to 0. Red line above graph indicates the time the laser was on. The black line above the graph indicates the average response duration for all traces shown. ***D***, Averaged and scaled ΔF/F traces from each prehearing *Th^2A-CreER^*;GCaMP6f with different shades of blue per animal. Black line within graph is the average of all the animal average traces. ΔF/F at time of photoablation and until imaging restarts is set to 0. ***E***, An SD image of a prehearing *Drd2^Cre^*;GCaMP6f mouse cochlea after focal ablation of OHCs with 1000 iterations of 100% laser power with hand drawn ROIs overlaid. Responsive ROIs are outlined with yellow. Scale bars: 25 μm. ***F***, ΔF/F traces of responsive ROIs from ***E***. ΔF/F at time of photoablation and until imaging restarts is set to 0. ***G***, Averaged and scaled ΔF/F traces from each prehearing *Drd2^Cre^*;GCaMP6f with different shades of green per animal. Black line within graph is the average of all the animal average traces. ΔF/F at time of photoablation and until imaging restarts is set to 0.

Following ablations that caused visible tissue damage, at least one type II neurite within the field of view experienced a transient increase in fluorescence in prehearing tissue 58% (26/45) of the time [neural response rate is defined as # photoablation with neural response/(# photoablation with neural response + # photoablation without neural response); Table 2]. Figure 3 shows the response of prehearing, apical and basal type II afferents following photoablation using *Th^2A-CreER^*;GCaMP6f and *Drd2^Cre^*;GCaMP6f mice, respectively. An example of a photoablation in the apex of a prehearing, *Th^2A-CreER^*;GCaMP6f animal is shown in Figure 3*A*. Figure 3*B* highlights the ROIs used in analysis of type II afferent segments where each yellow, outlined region responded to the photoablation. The responses to damage in prehearing, type II afferents from *Th^2A-CreER^*;GCaMP6f or *Drd2^Cre^*;GCaMP6f mice typically decreased monotonically from the first image after laser ablation (Fig. 3*C*,*D*,*F*,*G*). The overall time from when the ΔF/F first rose above 3 SDs of the baseline to within 3 SDs of the steady state value was defined as the response duration and is represented by the black bar above ΔF/F plots. The average response duration for prehearing type II afferents was 45.5 ± 25.0 s (Fig. 3*C*,*D*). Considering the median gap time of 7.2 s in imaging during and after photoablation, these response times are similar in length to the membrane currents (90% decay time of 58.5 s) measured by tight-seal intracellular recordings from type II afferents (Liu et al., 2015). This suggests that the GCaMP6f fluorescence closely follows the electrical response of the type II afferent to acute damage.

**Table 2:** *Th^2A-CreER^* and *Drd2^Cre^*;GCaMP6f*^fl^*^/^*^fl^* videos

	Prehearing	Mature	*Th* (apex)	*Drd2* (base)	External	PPADS	Total
Total number of videos (#)	117	144	109	152	204	57	261
# With neurons in view	100	116	90	126	173	43	216
Total number of mice	21	24	18	27	45	23	45
Number of mice with neurons in view	20	23	18	25	43	23	43
# Photoablation with neural response	26	9	11	24	26	9	35
# Photoablation without neural response	19	34	24	29	39	14	53
# Photoablation with no damage	11	34	15	30	40	5	45
# With NGAF	73	51	48	76	97	27	124
# Without NGAF	44	93	61	76	107	30	137

Number of videos for *Th^2A-CreER^* and *Drd2^Cre^*;GCaMP6f mice by category. Individual cells represent the number of videos or mice as defined by the leftmost column for the condition described in the topmost row. Videos for *Th^2A-CreER^*;GCaMP6f were taken from apical sections of the cochlea, whereas videos from *Drd2^Cre^*;GCaMP6f are from basal sections of the cochlea. Videos recorded when the tissue is bathed in external solution without the presence of PPADS are designated external and ones with PPADS in the bath are designated as PPADS. NGAF refers to NGAF. To calculate the neuronal response rate, take the value of # photoablation with neural response and divide it by the sum of the values for # photoablation with neural response and # photoablation without neural response. To calculate the NGAF response rate, take the value of # with NGAF and divide it by the sum of the values for # with NGAF and # without NGAF.

**Figure 4.**
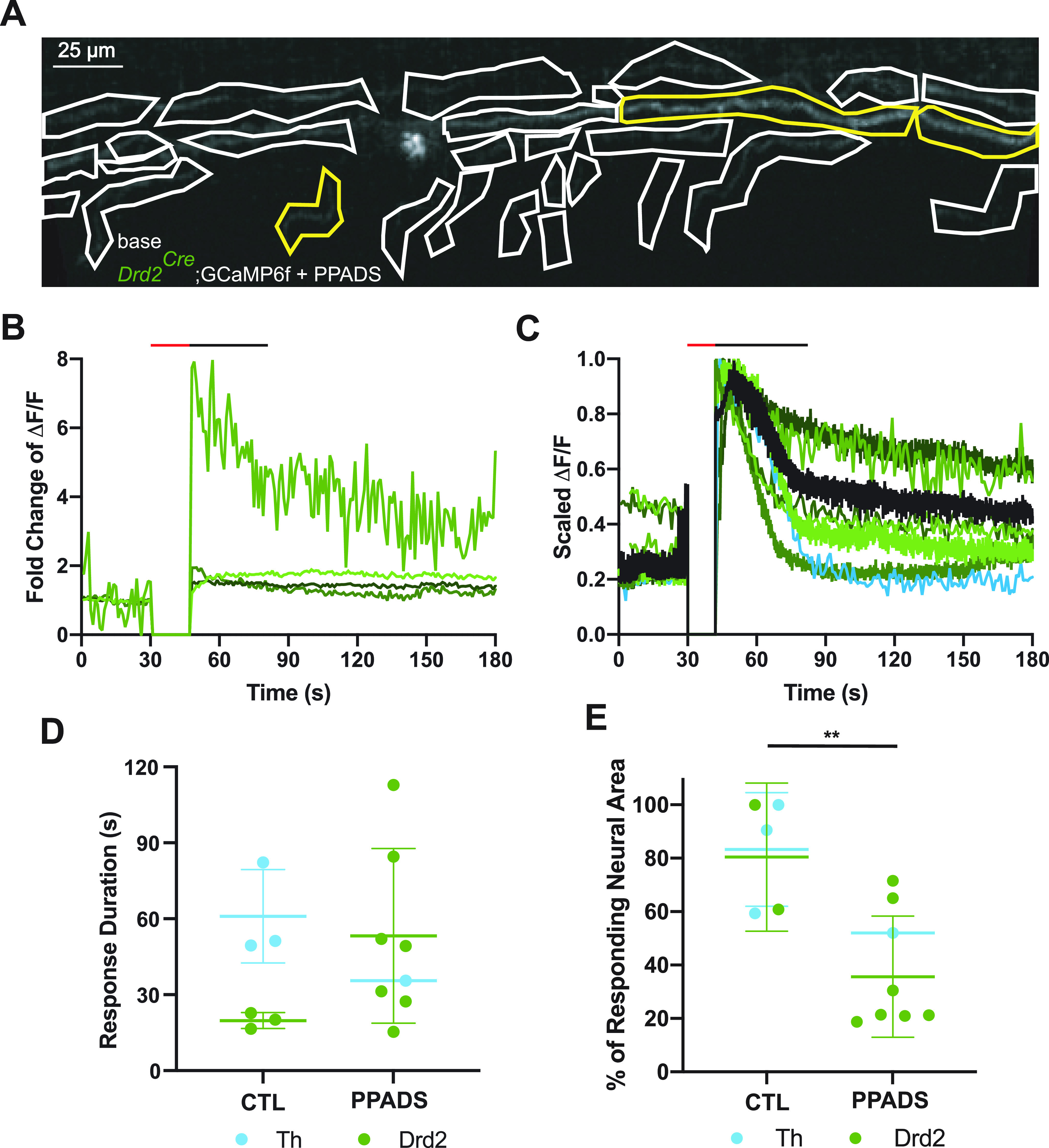
ATP blockade prevents the response of distant type II cochlear afferent fibers. ***A***, An SD projection image of a prehearing *Drd2^Cre^*;GCaMP6f with 100 μm PPADS in the extracellular solution with hand drawn ROIs. Yellow outlined ROIs represent responsive ROIs. Scale bar: 25 μm. ***B***, ΔF/F traces of the transiently responding ROIs in ***A***. ΔF/F at time of photoablation and until imaging restarts is set to 0. Red line above graph indicates the time the laser was on. The black line above the graph indicates the average response duration for all traces shown. ***C***, Averaged and scaled ΔF/F traces of responses from each prehearing *Drd2^Cre^*;GCaMP6f animal when 100 μm PPADS was in the extracellular solution with green colors representing different *Drd2^Cre^*;GCaMP6f animal and blue representing *Th^2A-CreER^*;GCaMP6f. Black line is the average of all the animal average traces. ΔF/F at time of photoablation and until imaging restarts is set to 0. Red line above graph indicates the time the laser was on. The black line above the graph indicates the average response duration for all traces shown. ***D***, Histogram of the response duration in seconds from the onset of photoablation to the time the response returns within 3 SDs of the steady state from prehearing animals with 100 μm PPADS and without 100 μm PPADS (CTL) in the extracellular solution. Blue dots represent *Th^2A-CreER^*;GCaMP6f animals and green dots represent *Drd2^Cre^*;GCaMP6f animals. Horizontal bar and vertical line represent average and SD for each group. ***E***, Histogram of the proportion of the area of responding neural ROIs to total area of neural ROIs for recordings from prehearing mice in 100 μm PPADS and without 100 μm PPADS (CTL) in the extracellular solution. Blue dots represent *Th^2A-CreER^*;GCaMP6f animals and green dots represent *Drd2^Cre^*;GCaMP6f animals. Horizontal bar and vertical line represent average and SD for each group; **p = 0.00011.

Transient calcium events occurred in type II afferents of the apex (*Th^2A-CreER^*;GCaMP6f animals; Fig. 3*A–D*) and base (*Drd2^Cr^*^e^;GCaMP6f animals; Fig. 3*E–G*) of the cochlea. Although these particular examples have markedly different time courses; overall, there were no significant differences in response probability or time course as a function of cochlear position. For all videos recorded from prehearing animals, the proportion of videos with at least one, visible, responding neuron was similar for both classes of neurons (neural response rate; Table 2; *Th* 11/35 videos vs *Drd2* 24/53 videos; *p* = 0.099, generalized mixed model) as were the response durations (Fig. 4*E*; *Th*: 54.6 ± 9.7s vs *Drd2*: 43.2 ± 32.5 s; *p* = 0.34, linear mixed model Satterthwaite’s method). Therefore, all following reported averages will reflect pooled *Th* and *Drd2* data, although the effect of genotype was always factored into statistical analyses.

### ATP contributes to the damage response of prehearing type II afferents

ATP is a major contributor to the response of type II cochlear afferents to focal OHC ablation (Liu et al., 2015). Blocking purinergic receptors with 100 μm PPADS, a generic P2X and partial P2Y receptor antagonist, greatly reduced the duration, but not the peak amplitude of a damage-evoked inward current in apical prehearing type II cochlear afferents (Liu et al., 2015). Using calcium imaging, bath applied 100 μm PPADS did not prevent neuronal responses to photoablation (Fig. 4*A–C*) and had no significant effect on the probability of a type II GCaMP6f response to damage within the field of view (neural response rate, Table 2; PPADS 8/12 videos vs control 20/31 videos). This is consistent with the ability of 100 μm PPADS to abbreviate but not completely block the damage-evoked membrane current in type II cochlear afferents (Liu et al., 2015). Also, 100 μm PPADS did not significantly alter the duration of the calcium signal in prehearing type II afferents (Fig. 4*D*; no PPADS: 40.4 ± 25.5 s vs PPADS 48.2 ± 31.5 s, *n* = 14 videos, *N* = 8 mice). However, 100 μm PPADS did significantly restrict the extent of activation of type II afferents within the field of view. This was measured as the summed area of responding type II ROIs as a fraction of the area of all type II ROIs in the field of view, hereafter referred to as neural response area (Fig. 4*E*; control: 62.7 ± 26.0% vs PPADS: 37.7 ± 21.8%, *p* = 0.00011, df = 5.34, *n* = 15 videos, *N* = 9 mice, linear mixed model Satterthwaite’s method). This observation supports the hypothesis that ATP release among surrounding supporting cells promotes the spatial and temporal spread of the response to damage (Gale et al., 2004; Liu et al., 2015; Sirko et al., 2019).

### NGAF reflects epithelial cell activity and depends on ATP

**Figure 5.**
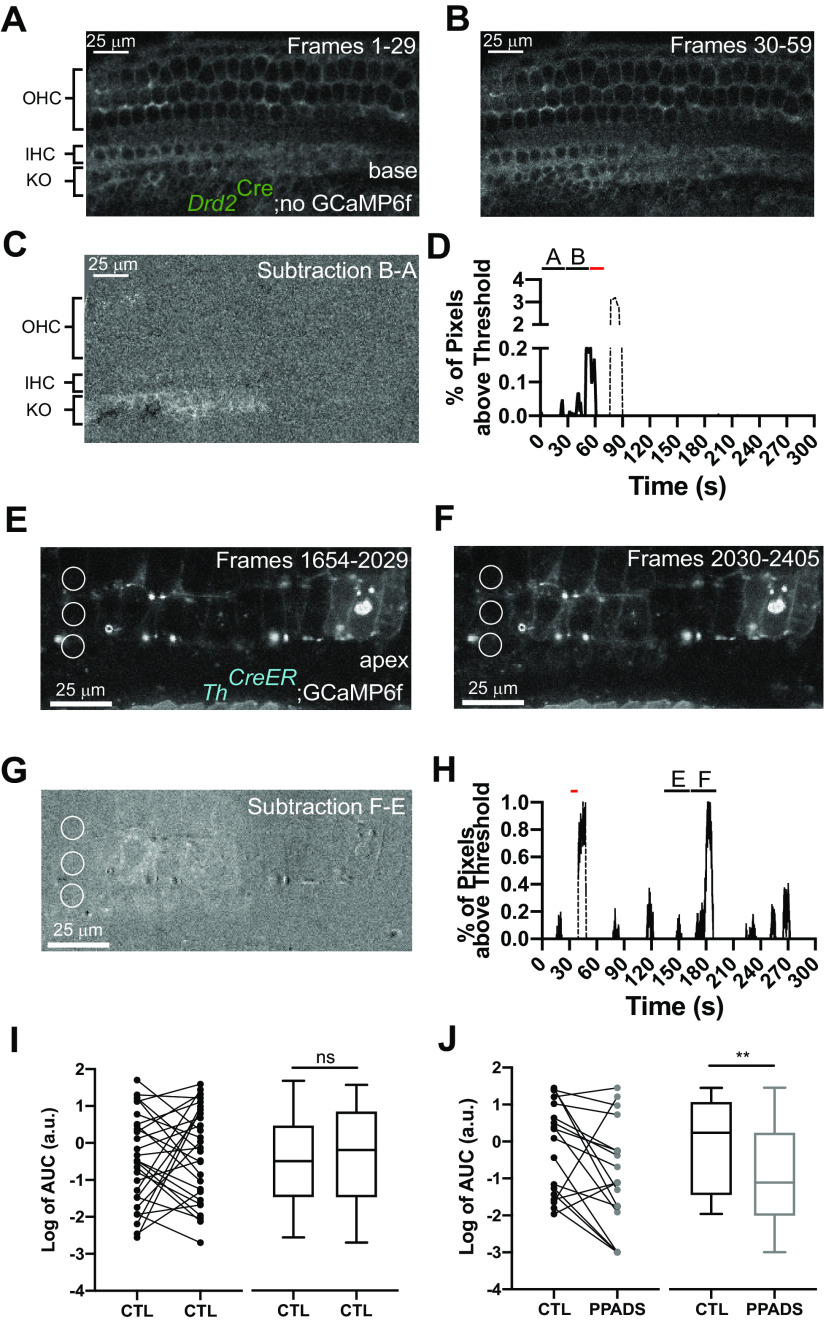
NGAF is PPADS sensitive and can occur spontaneously. ***A***, SD image of a recording from a prehearing *Drd2^Cre^* animal with no GCaMP6f expression. ***B***, Same image as ***A*** but during the frames when spontaneous increase in fluorescence occurred. ***C***, Image generated by subtracting ***B*** from ***A***. Scale bars: 25 μm. Brackets on the side refer to the location of OHCs, IHCs, and Kölliker’s organ (KO) in the image. ***D***, Percent of total pixels above an arbitrary threshold from spontaneous events from the recording in ***A***, ***B***. Black bars represent the time of the images in ***A***, ***B***. Red bar denotes the time when photoablation occurred. The NGAF deflection immediately following photoablation is disregarded from analysis and is shown as dashed lines. ***E***, SD image of a recording from a mature *Th^2A-CreER^*;GCaMP6f animal. ***F***, Same image as ***E*** but during the frames when spontaneous increase in fluorescence occurred. ***G***, Image generated by subtracting ***F*** from ***E***. Scale bars: 25 μm. ***H***, Percent of total pixels above an arbitrary threshold from spontaneous events from the recording in ***E***, ***F***. Black bars represent the time of the images in ***A***, ***B***. Red bar denotes the time when photoablation occurred. The NGAF deflection immediately following photoablation is disregarded from analysis and is shown as dashed lines. ***I***, ***J***, left, Paired dot and (right) box and whisker plot of log transformed area under the curve values from paired NGAF recordings before and after the addition of control extracellular solution (***I***) or 100 μm PPADS in the extracellular solution (***J***); **p = 0.0071.

Previous studies used membrane permeant dyes to describe ATP-dependent calcium waves propagating through cochlear supporting cells after acute tissue damage (Anselmi et al., 2008; Gale et al., 2004; Sirko et al., 2019). In the present work, temporal and spatial variations in fluorescence were evident even in tissue that did not express the indicator GCaMP6f; referred to henceforth as NGAF events, presumably resulting from tissue-dependent changes in the optical path. NGAF events appeared transiently in large swaths of tissue either in the greater epithelial ridge region in pre-hearing animals (Fig. 5*A–C*) or in the area of the OHCs mature animals (Fig. 5*E–G*). Given their location and duration, NGAF events are likely to be the consequence of ATP-dependent waves among supporting cells. This activity was observed originally by differential interference contrast (DIC) imaging because of the physical shrinkage and increased extracellular space from ionic flux and associated water loss, termed crenation (Tritsch et al., 2007, 2010; Babola et al., 2020). The sensitivity of the two-photon microscope used in the present experiments enables detection of crenations among supporting cell tissue as the transmitted fluorescence varies with optical density.

NGAF activity occurred spontaneously (Fig. 5*A–C*) or immediately following focal OHC ablation (Fig. 5*E–G*). Both spontaneous and evoked NGAF events occurred in a mouse with no GCaMP6f expression in any cell type under similar recording conditions (Fig. 5*A–D*; Movie 1). Negative immunostaining for GCaMP6f additionally confirmed that NGAF is not because of ectopic expression of GCaMP6f in non-neuronal cells of the organ of Corti.

Movie 1. Photoablation causes NGAF responses in an animal without GCaMP6f fluorescence. Video created from images taken every second before and after a photoablation in a prehearing animal with no GCaMP6f expression. Video has been sped up 20 from raw footage. The word ablation appears for 1 s just after the photoablation occurs in the top left corner. Before the photoablation, slight fluorescence changes are seen in the area of the greater epithelial region corresponding to crenation events. After photo ablation, NGAF is observed in the OHC region. 10.1523/ENEURO.0383-21.2021.video.1

The magnitude of NGAF activity was calculated as the area under the curve of deflections in brightness over an arbitrary threshold (Fig. 5*D*,*H*). The presence of 100 μm PPADS caused no significant effect on NGAF magnitude when pooling across animals as measured by the area under the curve of NGAF activity (data not shown). This may be because of the large variability observed across animals. To circumvent the differences between animals, paired recordings were compared in the same cochlea for focal ablations at different locations. Shifting the location of focal ablation did not have a significant effect on the NGAF magnitude as the magnitude was just as likely to increase as decrease when ablation was repeated in control solutions (Fig. 5*I*; *p* = 0.66, *N* = 28 cochleas, paired Wilcoxon signed-rank test). In contrast, paired videos where the second ablation occurred with 100 μm PPADS in the bath had a reduced NGAF magnitude compared with preceding activity 15 out of 19 times (Fig. 5*J*; *p* = 0.0071, *N* = 19 cochleas, paired Wilcoxon signed-rank test). Overall, the dynamics, location, and ATP-dependence of NGAF activity support the interpretation that these are another sign of the previously reported activity waves among cochlear supporting cells (Gale et al., 2004; Ceriani et al., 2019; Sirko et al., 2019).

### Generation and verification of the *Tac1^Cre^*;GCaMP6f mouse model

**Figure 6.**
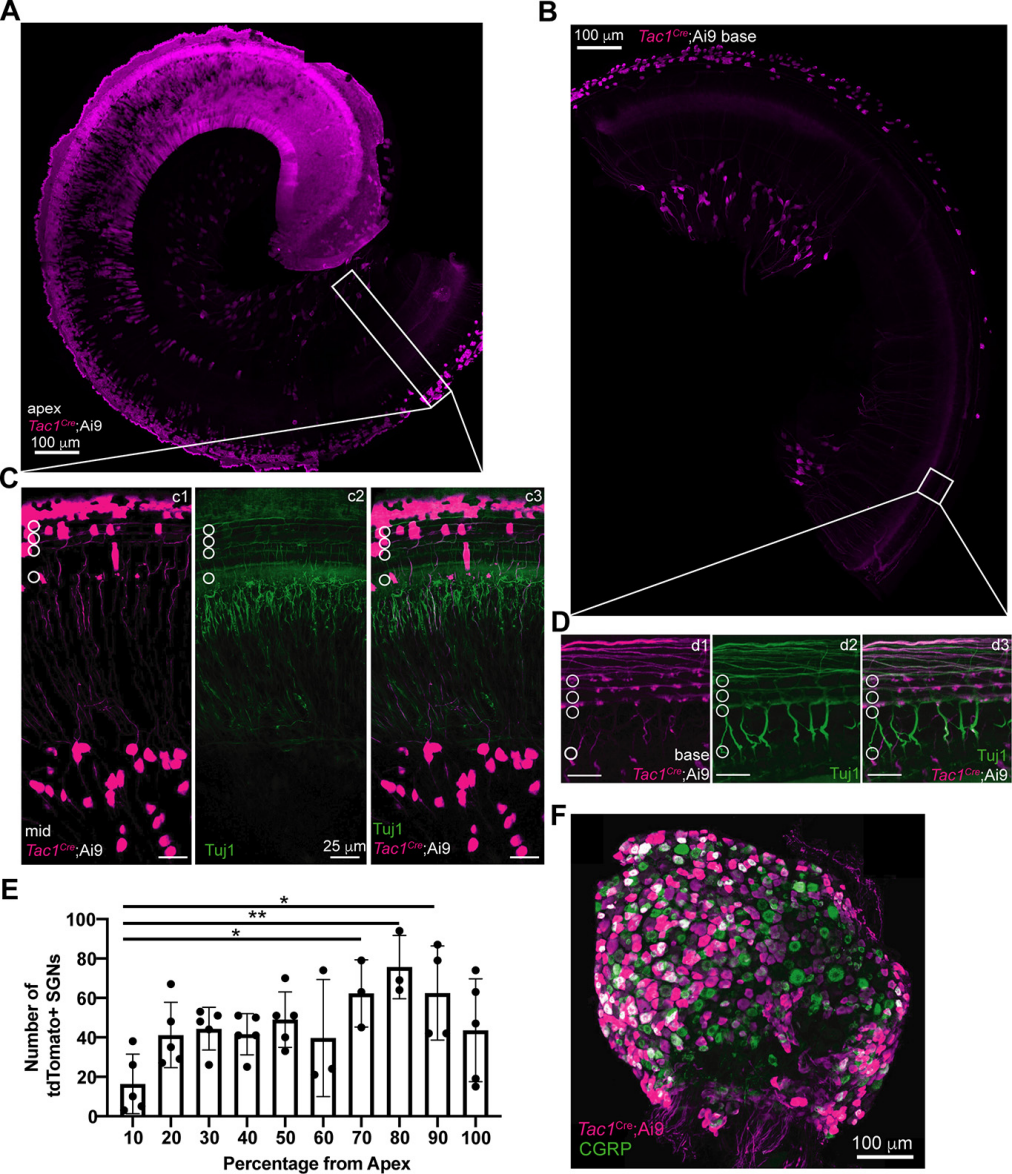
Expression of GCaMP6f by *Tac1^Cre^* driver labels type II afferents along the tonotopic axis and has broad supporting cell expression in the apex. ***A***, ***B***, 5× magnification confocal images of prehearing *Tac1^Cre^*;Ai9 mouse apical (***A***) and basal segments of cochlea. Scale bar: 100 μm. ***C***, ***D***, 25× magnification confocal images of a prehearing *Tac1^Cre^*;Ai9 mouse middle section of cochlea spanning the spiral ganglion and organ of Corti (***C***) and basal organ of Corti (***D***) representing the white boxes in ***A***, ***B***, respectively. ***c1***, ***d1***, Magenta channel alone (anti-tdTomato, i.e., *Tac1^Cre^*-expressing cells). ***c2***, ***d2***, Green channel alone (anti-Tuj1, pan-neuronal marker). ***c3***, ***d3***, Overlay of the two channels. Neurons that show co-localized expression of tdTomato and Tuj1 appear white. Scale bar: 25 μm. ***E***, Histogram of spiral ganglion cell somata count with 10% bins across the tonotopic axis; *p < 0.05, **p < 0.01. 10% versus 70%: *p* = 0.044; 10% versus 80% *p* =0.0033; 10% versus 90%: *p* = 0.020. ***F***, 5× magnification confocal images of a *Tac1^Cre^*;Ai9 dorsal root ganglion (anti-tdTomato, *Tac1^Cre^*-expressing cells, magenta; anti-CGRP, C-fiber marker, green). Neurons that show co-localized expression of tdTomato and CGRP appear white. Scale bar: 100 μm.

Although NGAF activity corresponds to supporting cell activity, it is not as bright or distinct as the fluorescence emitted from GCaMP6f expressing cells. A better system would exploit GCaMP6f fluorescence in both supporting cells and type II cochlear afferents. This proved possible using a peculiarity in expression of the *Tac1* gene by cochlear tissue. Alternative splicing of the *Tac1* gene leads to the production of four different neuropeptides, including substance P (Krause et al., 1987). *Tac1* is expressed by peptidergic somatosensory neurons in the dorsal root ganglia (Fig. 6*F*; Weissner et al., 2006; Kestell et al., 2015). RNA-seq from cochlear afferents suggested that type II cochlear afferents and one subtype of type I cochlear afferents could express *Tac1* (Shrestha et al., 2018). *Tac1^Cre^* mice crossed with Ai9 (floxed tdTomato) reporter mice exhibited a unique expression pattern: whereby nearly every hair cell and supporting cell in the cochlear apex expressed tdTomato (Fig. 6*A*), but with a progressive absence of expression in epithelial cells toward the cochlear base (Fig. 6*B*). In sharp contrast, a subset of SGNs expressed tdTomato throughout the cochlear length except at the extreme apical tip where SGNs are few (Vyas et al., 2019; Fig. 6*E*; df = 9, f value = 3.104, *p* = 0.0082, *N* = 5 animals, one-way ANOVA). Excluding the first 10% of the cochlea, there were no significant differences in SGN expression of tdTomato among the remaining areas along the cochlear spiral (df = 8, f value = 1.605, *p* = 0.17, *N* = 5 animals, one-way ANOVA). The total number of SGNs expressing tdTomato was equivalent to all type II cochlear afferents plus a minority of type I cochlear afferents. Type II afferent identity was confirmed by taking higher magnification confocal images of *Tac1^Cre^*; Ai9 cochleas in the OHC region. Many of the fluorescent fibers turned basally, ran parallel underneath the OHCs (Fig. 6*C*,*D*) and projected bouton endings to OHCs (Fig. 6*d1*,*d3*), confirming their type II afferent identity.

### Type II afferent and epithelial cell calcium activity in *Tac1^Cre^*;GCaMP6f mice

**Figure 7.**
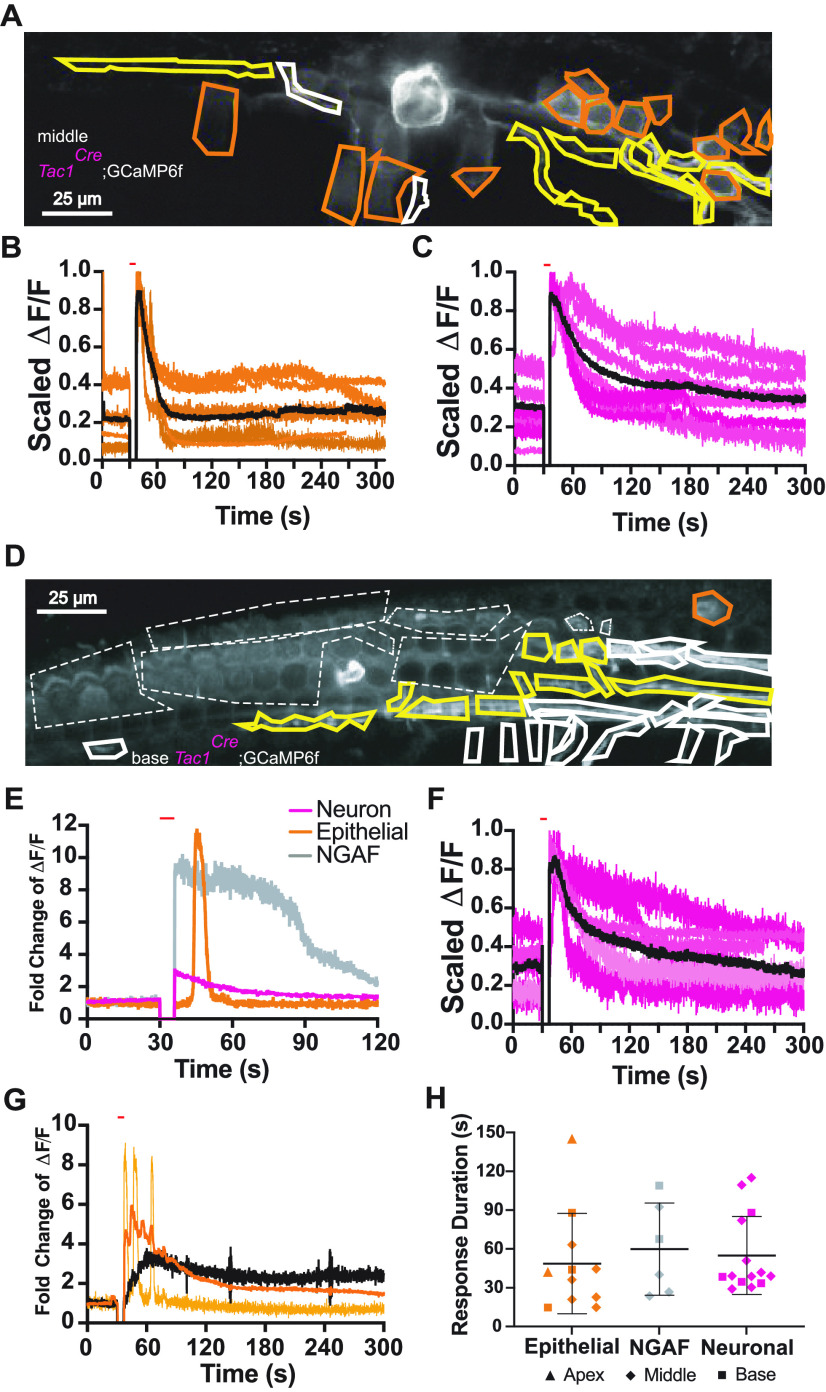
Expression of GCaMP6f by *Tac1^Cre^* driver reveals type II afferent activity in the base as well as epithelial cell calcium activity in the middle section of the cochlea. ***A***, SD image of a focal ablation in the middle section of a prehearing *Tac1^Cre^*;GCaMP6f mouse cochlea with hand drawn ROIs around segments of neurons and epithelial cells. Scale bar: 25 μm. Yellow, outlined neuronal segments represent neurons that significantly respond to the stimulus and orange, outlined epithelial cells represent epithelial cells that significantly respond. ***B***, ΔF/F traces from responding epithelial cell ROIs in ***A***. Black line is the average of all traces. ΔF/F at time of photoablation and until imaging restarts is set to 0. ***C***, Averaged and scaled ΔF/F traces for neuronal responses (shades of magenta) from the middle sections of *Tac1^Cre^*;GCaMP6f mice. Black line is the average of the average traces from each recording. ΔF/F at time of photoablation and until imaging restarts is set to 0. ***D***, SD image of a focal ablation in the basal section of a prehearing *Tac1^Cre^*;GCaMP6f mouse cochlea with hand drawn ROIs around segments of neurons, epithelial cells, and NGAF activity. Scale bar: 25 μm. Yellow outlined neuronal segments represent neurons that significantly responded to the stimulus, orange outlined ROIs represent epithelial cells that significantly respond, and dashed, white ROIs represent areas with NGAF activity. ***E***, Average ΔF/F traces from responding ROIs in ***D***. NGAF trace shown in gray, epithelial cell response in orange, and average neuron response in magenta. ΔF/F at time of photoablation and until imaging restarts is set to 0. ***F***, Averaged and scaled ΔF/F traces for neuronal responses (shades of magenta) from the basal sections of *Tac1^Cre^*;GCaMP6f mice. Black line is the average of the average traces from each recording. ΔF/F at time of photoablation and until imaging restarts is set to 0. ***G***, Average ΔF/F traces from responding ROIs of another prehearing *Tac1^Cre^*;GCaMP6f mouse. An example individual epithelial cell calcium response in yellow, the average epithelial cell response in orange, and average neuron response in black. ΔF/F at time of photoablation and until imaging restarts is set to 0. ***H***, Histogram of the individual response durations from NGAF, epithelial cell and neuron ROIs across all *Tac1^Cre^*;GCaMP6f mice across the cochlea. Triangles represent responses from apical cochlea sections, diamonds from middle cochlea sections, and squares from basal cochlea sections. Magenta represents neurons, orange represents epithelial cells, and gray represents NGAF activity.

*Tac1^Cre^* mice were crossed with floxed GCaMP6f mice to express GCaMP6f in all cell types that have expressed Tac1 at any point in development. In the apex of the cochlea, calcium imaging in prehearing mice recapitulates observations of spontaneous crenations and widespread calcium responses to damage (data not shown; Gale et al., 2004; Tritsch et al., 2007). The most informative *Tac1* expression pattern, however, is in the middle turn of the cochlea where there is stochastic expression in epithelial cells, providing concurrent viewing of type II cochlear afferent neurites through the gaps in epithelial cell expression (Fig. 7*A*). Analysis of videos in this region of the cochlea (Movie 2) facilitates the simultaneous observation of calcium events in epithelial cells (Fig. 7*B*) and type II cochlear afferents (Fig. 7*C*). Prehearing type II afferents labeled by *Tac1^Cre^* are capable of responding to local photoablation (neural response rate, Table 3; 22/36 videos). The average response duration for epithelial cells and neurons statistically indistinct (Fig. 7*H*).

**Table 3:** *Tac1^Cre^*;GCaMP6f*^fl^*^/^*^fl^* videos

	Prehearing	Mature	Apical	Middle	Basal	External	PPADS	Total
Total number of videos (#)	36	38	14	50	10	58	16	74
Total number of mice	6	6	9	10	6	12	8	12
# Photoablation with response	14	8	3	14	5	19	3	22
# Photoablation without neural response	6	8	2	11	1	7	7	14
# Photoablation no damage	4	9	2	9	2	12	1	13
Epithelial cell response	24	4	11	14	3	23	5	28
# With NGAF	2	13	0	11	4	10	5	15

Number of videos for *Tac1^Cre^*;GCaMP6f mice by category. Individual cells represent the number of videos or mice as defined by the leftmost column for the condition described in the topmost row. Videos for *Th^2A-CreER^*;GCaMP6f were taken from apical sections of the cochlea, whereas videos from *Drd2^Cre^*;GCaMP6f are from basal sections of the cochlea. Videos recorded when the tissue is bathed in external solution without the presence of PPADS are designated external and ones with PPADS in the bath are designated as PPADS. NGAF refers to NGAF. To calculate the neuronal response rate, take the value of # photoablation with neural response and divide it by the sum of the values for # photoablation with neural response and # photoablation without neural response. To calculate the NGAF response rate, take the value of # with NGAF and divide it by the sum of the values for # with NGAF and # without NGAF.

**Table 4:** Noise-exposed and control videos

	NE	Control	*Th* (apex)	*Drd2* (base)	External	PPADS	Total
Total number of videos (#)	74	79	108	45	127	26	153
# With neurons in view	52	62	89	25	93	21	114
Total number of mice	12	13	18	7	25	14	25
Number of mice with neurons in view	11	12	18	5	25	14	23
# Photoablation with neural response	10	7	17	0	16	1	17
#Photoablation without neural response	21	27	31	17	40	8	48
# Photoablation no damage	4	6	8	2	7	3	10
# With NGAF	37	26	41	22	54	9	63
# Without NGAF	37	53	67	23	73	17	90
# With delayed response	16	10	23	3	25	1	26

Number of videos for *Th^2A-CreER^* and *Drd2^Cre^*;GCaMP6f noise-exposed mice and their control littermates by category. Individual cells represent the number of videos or mice as defined by the leftmost column for the condition described in the topmost row. Videos for *Th^2A-CreER^*;GCaMP6f were taken from apical sections of the cochlea whereas videos from *Drd2^Cre^*;GCaMP6f are from basal sections of the cochlea. Videos recorded when the tissue is bathed in external solution without the presence of PPADS are designated external and ones with PPADS in the bath are designated as PPADS. NGAF refers to NGAF. To calculate the neuronal response rate, take the value of # photoablation with neural response and divide it by the sum of the values for # photoablation with neural response and # photoablation without neural response. To calculate the NGAF response rate take the value of # with NGAF and divide it by the sum of the values for # with NGAF and # without NGAF. To calculate the delayed response rate, take the value of # with delayed response and divide it by the sum of the values for # photoablation with neural response and # photoablation without neural response.

Movie 2. Neuronal and epithelial cells respond to photoablation in the middle turn of a *Tac1^Cre^*;GCaMP6f animal. Video created from images taken every 80 ms before and after a photoablation. Video has been sped up 20× from raw footage. The word ablation appears in the top left corner of the video frame for 4 s after the photoablation event occurs. Neurons and epithelial cells increase in fluorescence intensity after the photoablation. 10.1523/ENEURO.0383-21.2021.video.2

When examining the base of the cochlea, the GCaMP6f expression is mostly restricted to neurons (Fig. 7*D*). Thus, NGAF response duration is used as a measure of epithelial response to photoablation. The response duration of neurons, NGAF, and epithelial cells are statistically insignificant (Fig. 7*H*) Individual epithelial cells may have a staggered, short (Fig. 7*E*) or repeating (Fig. 7*G*) response to photoablation. However, when averaged, the total response duration for all epithelial cells is not distinguishable from the response durations of neurons or NGAF (Fig. 7*H*; NGAF: 59.9 ± 35.6 s, type II GCaMP6f: 50.1 ± 37.3 s, vs epithelial cell GCaMP6f: 54.2 ± 29.2 s, *p*_epithelial cell_ = 0.91 df = 24.2, *p*_NGAF_ = 1.0 df = 22.9, *p*_type II_ = 1.0 df = 19.2; *n* = 20 videos, *N* = 14 cochleas, linear mixed model Satterthwaite’s method with Bonferroni correction). This suggests that NGAF activity is not an exact readout of calcium signaling within individual epithelial cells; but, instead, demonstrates the summed activity of epithelial tissue.

### Type II cochlear afferent responses to tissue ablation are less frequent and longer lasting in mature cochleas

Calcium imaging of otic capsule preparations enabled study of fully mature tissue for which intracellular recording is difficult. Mature (between 6 and 10 weeks of age) type II cochlear afferents were capable of responding to focal OHC ablation; however, responses occurred about one third as often as in cochleas from prehearing animals. This reduced response probability to damage was based only on those cases where OHC damage and type II afferent dendrites were clearly visible (neural response rate, Table 2; mature: 9/43 vs prehearing: 26/47, *p* = 0.00046, *N* = 22 cochleas, generalized mixed model). In mature cochleas damage occurred less reliably. The decrease in response rate to damage could not be explained by a decrease in the amount of damage caused by the focal laser ablations. When it occurred, the area of damage was not significantly different in size between the prehearing and mature animals. Rather, independent of age, scar caused by the laser was significantly larger in cases where the damage caused type II afferent responses (with response: 305.8 ± 251.6 μm^2^ vs without response: 209.8 ± 182.7 μm^2^, *p* = 0.009, df = 118.4, *n* = 153 videos, *N* = 72 cochleas, linear mixed model, Satterthwaite’s method). This points to a correlation between the amount of damage and the neuronal response, or perhaps a threshold for evoking neuronal responses.

**Figure 8.**
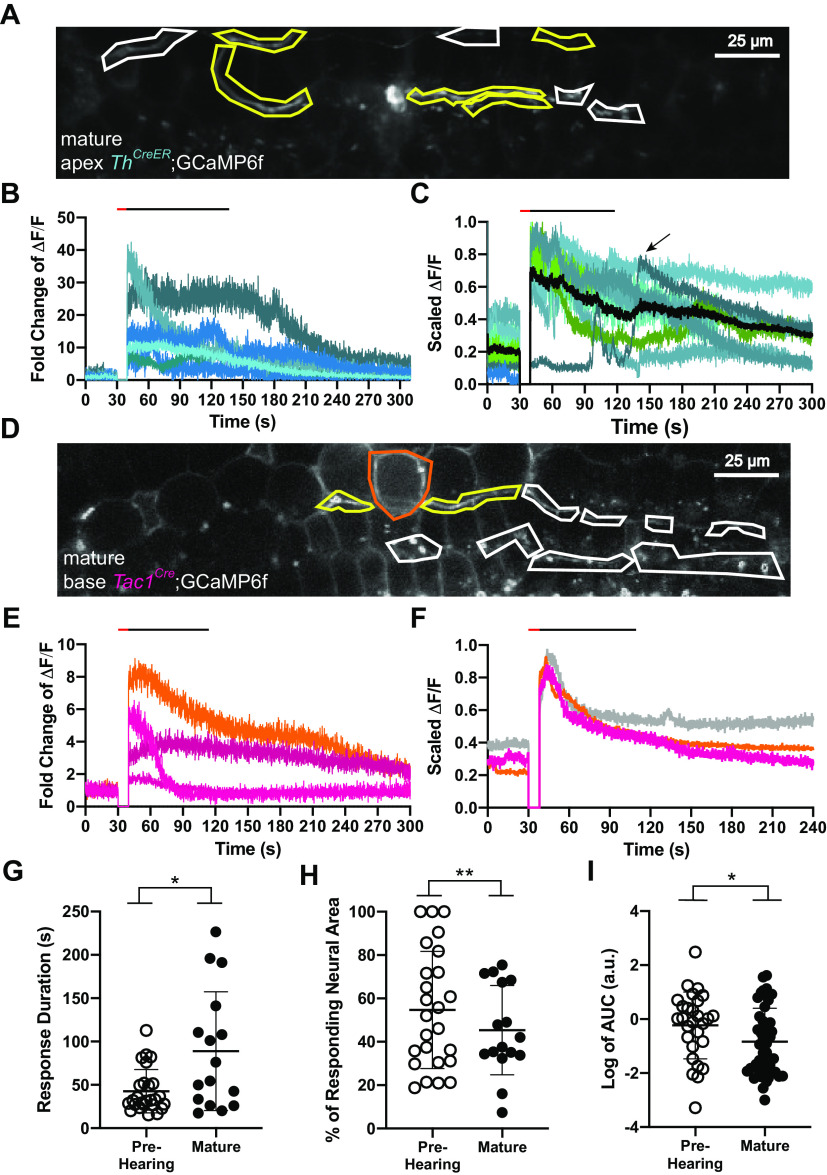
Mature organs of Corti have reduced spread of NGAF and neuronal activation. ***A***, SD image of a mature *Th^2A-CreER^*;GCaMP6f mouse apical cochlea after focal ablation with hand drawn ROIs around segments of neurons. Yellow, outlined neuronal segments represent neurons that significantly responded to the stimulus. Scale bar: 25 μm. ***B***, ΔF/F traces from responding ROIs in ***A***. ΔF/F at time of photoablation and until imaging restarts is set to 0. ***C***, Averaged and scaled traces of all mature *Th^2A-CreER^*;GCaMP6f and *Drd2^Cre^*;GCaMP6f recordings arrow points to an example trace with a delayed response. Black line is the average of the average traces from each recording. ΔF/F at time of photoablation and until imaging restarts is set to 0. ***D***, SD image of a mature *Tac1^Cre^*;GCaMP6f mouse basal cochlea after focal ablation with hand drawn ROIs around segments of neurons. Yellow, outlined neuronal segments represent neurons that significantly responded to the stimulus and orange, outlined segment for the responding epithelial cell. Photoablation occurred within orange outline. Scale bar: 25 μm. ***E***, ΔF/F traces from responding ROIs in ***D***. Magenta traces represent neural ROIs and orange trace represents the epithelial ROI. ΔF/F at time of photoablation and until imaging restarts is set to 0. ***F***, Averaged and scaled neuronal (magenta), epithelial (orange), and NGAF (gray) traces of all mature *Tac1^Cre^*;GCaMP6f recordings. ΔF/F at time of photoablation and until imaging restarts is set to 0. ***G–I***, Dot plots of (***G***) response duration from the observed peak to the time the response returns within 3 SDs of the steady state, *p = 0.020 and *p* = 0.041 for the interaction (mature), (***H***) the proportion of area of responding neural ROIs to total area of neural ROIs, **p = 0.0030 and *p* = 0.0036 for the interaction, and (***I***) the log transformed values of the area under the curve of spontaneous increases in fluorescence from mature mice, *p = 0.0346.

Type II afferents from mature *Th^2A-CreER^*^,^ and *Drd2^Cre^* (Fig. 8*A–C*), as well as *Tac1^Cre^* (Fig. 8*D–F*) animals expressing GCaMP6f responded to focal ablation of OHCs. As before, ROIs with colored outlines in each SD plot represent the location of type II afferent dendrites or epithelial cells that respond to the focal ablation (Fig. 8*A*,*D*). The ΔF/F traces of each responsive element in each example are shown in Figure 8*B*,*E* and the averages for each recording in Figure 8*C*,*F*. The response duration was significantly longer in mature compared with prehearing animals (Fig. 8*G*; mature: 73.5 ± 53.8s vs prehearing: 42.5 ± 25.0 s, *p* = 0.020, df = 18, *n* = 42 videos, *N* = 27 cochleas, linear mixed model Satterthwaite’s method). The neuronal response area was significantly reduced in mature compared with prehearing responses (Fig. 8*H*; prehearing: 63 ± 26% vs mature: 41 ± 15%, *p* = 0.0030, df = 23.5, *n* = 35 videos, *N* = 14 cochleas, linear mixed model Satterthwaite’s method). Furthermore, the frequency of NGAF activity was reduced in mature tissue compared with prehearing tissue [NGAF response rate defined as # with NGAF/(# with NGAF + # without NGAF); Table 2; mature: 51/144 vs prehearing: 73/117, *p* = 2.18 × 10^−6^, *N* = 52 cochleas, generalized mixed model]. Also, the NGAF magnitude in mature tissue decreased relative to that found in prehearing tissue (Fig. 8*I*; prehearing: 13.4 ± 56.9 a.u. vs mature: 3.3 ± 8.4 a.u., *p* = 0.0346, df = 31.2, *n* = 73 videos, *N* = 35 cochleas, linear mixed model Satterthwaite’s method). When NGAF activity did occur in mature tissue, it was situated mostly in the OHC region instead of the greater epithelial ridge as was the case in the prehearing animals (Fig. 5*E*; Sirko et al., 2019). Overall, while mature cochlear tissue still could respond to focal damage, such responses were less frequent, less extensive, but longer-lasting than those in the prehearing cochlea.

### Acoustic trauma enhances type II afferent and epithelial responses to focal ablation

**Figure 9.**
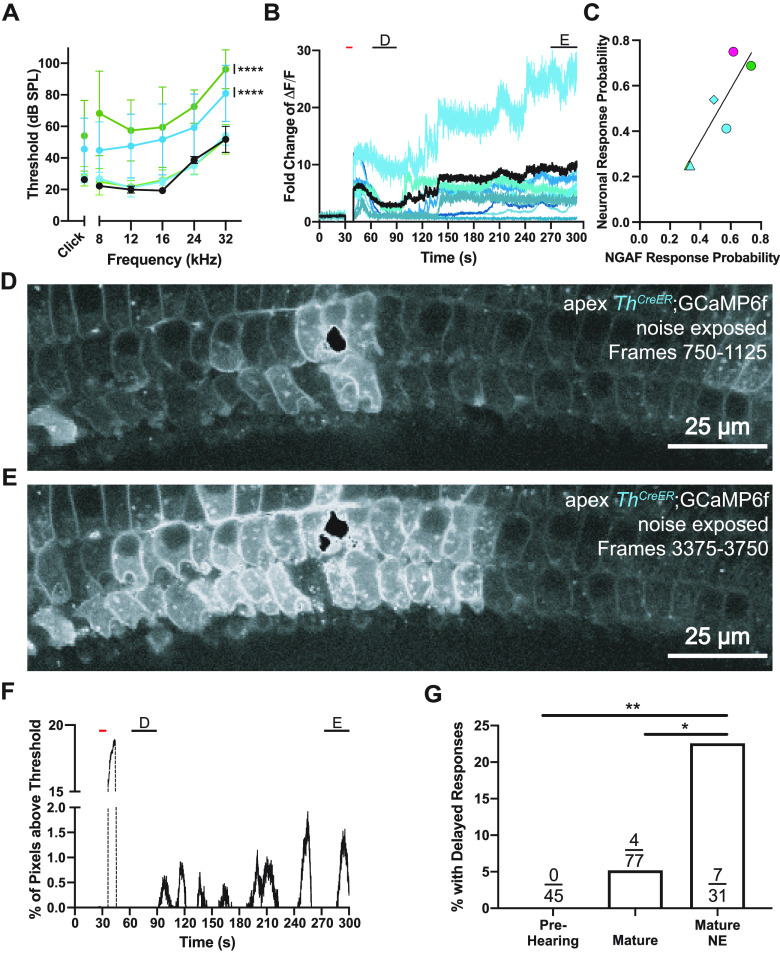
Previous acoustic trauma increases the chance for cochlear activity long after an acute damage stimulus has subsided. ***A***, Average ABR threshold measurements from noise-exposed *Th^2A-CreER^*;GCaMP6f and *Drd2^Cre^*;GCaMP6f mice and their control littermates 1 d before calcium imaging (7 or 21 d after acoustic trauma). Light cyan trace represents control *Th^2A-CreER^*;GCaMP6f mice, *N* = 8, and dark cyan represents noise-exposed *Th^2A-CreER^*;GCaMP6f mice, *N* = 10. Light green trace represents control *Drd2^Cre^*;GCaMP6f mice, *N* = 4, and dark green represents noise-exposed *Drd2^Cre^*;GCaMP6f mice, *N* = 4. Black trace represents two control wild-type (C57BL/6J) mice tested; ****p ≤ 0.0001. ***B***, ΔF/F traces from a representative *Th^2A-CreER^*;GCaMP6f recording 7 d after noise. Black line is the average of all the ROIs from this recording. ΔF/F at time of photoablation and until imaging restarts is set to 0. Red bar denotes time of photoablation. Black bars denote time windows used to generate images in ***D***, ***E***. ***C***, Correlation of neuronal response rate to NGAF response rate for each group of data. Color indicates genotype. Shape indicates age or condition. Cyan, *Th^2A-CreER^*;GCaMP6f; green, *Drd2^Cre^*;GCaMP6f; magenta, *Tac1^Cre^*;GCaMP6f. Circle, prehearing; triangle, mature; diamond, noise-exposed. ***D***, SD image of a mature 7 d postnoise exposure *Th^2A-CreER^*;GCaMP6f mouse apical cochlea 30 s after focal ablation for 30 s. ***E***, SD image of the mature 7 d postnoise exposure *Th^2A-CreER^*;GCaMP6f mouse apical cochlea from ***C***, several minutes after focal ablation for 30 s. Scale bars: 25 μm. ***F***, Percent of total pixels above an arbitrary threshold from spontaneous events from the recording in ***B–E***. Red bar denotes time of photoablation. Black bars denote time windows used to generate images in ***D***, ***E***. The NGAF deflection immediately following photoablation is disregarded from analysis and is shown as dashed lines. ***G***, Bar plot of the proportion of videos that have neural activity reemerge minutes after the photoablation in different conditions. Number of videos with delayed response over the number of videos with an ablation event displayed in each bar of the bar graph. CTL, control; NE, noise-exposed, *p = 0.037. Prehearing to noise-exposed, **p = 0.0036.

The present methods apply to type II afferents in older cochlear tissue, enabling the study of the effects of previous acoustic trauma on the acute damage responses. Mice were exposed to 2 h of 110 dB broadband noise, then compared with their control, untraumatized littermates 7 or 21 d later. No difference was observed between mice 7 or 21 d after noise exposure; therefore, data from these mice have been grouped together Table 4. Noise-exposed mice had a significant increase in ABR thresholds measured the day before calcium imaging experiments as compared with littermate controls and wild-type mice (Fig. 9*A*; two-way ANOVA, *Drd2*: *p* ≤ 0.0001, *F*_(2 DFn,42 DFd)_ = 49.59; *Th*: p ≤ 0.0001, *F*_(2 DFn,102 DFd)_ = 38.45). A unique feature of responding neuronal ROIs after noise exposure was a brief calcium transient to photoablation followed by a secondary, delayed response several minutes after the initial change in GCaMP6f fluorescence (Movie 3). ΔF/F traces of type II afferent dendrites in this example from a *Th^2A-CreER^*;GCaMP6f mouse reveal an initial response to the focal ablation followed by increases over the next few minutes (Fig. 9*B*). As in the previous prehearing and mature cochleas, NGAF activity was observed alongside neuronal responses and occasionally mirrored the delayed pattern seen in the neurons. Together, over the various genotypes, ages, and noise exposure statuses there was a significant correlation between the proportion of times we observed NGAF activity and neuronal responses to damage in all videos of each condition (Fig. 9*C*; *R*^2^ = 0.78, *p* = 0.020 *F*_(1 DFn,4 DFd)_ = 13.98). Figure 9*D*,*E* shows an example of the extent of both the neuronal and epithelial cell responses immediately following focal ablation (Fig. 9*D*) and at the end of the recording (Fig. 9*E*). Increases in epithelial cell fluorescence are visualized by measuring the increases in the percentage of pixels above an arbitrary brightness (Fig. 9*F*) and reveal the repetitive nature of the delayed NGAF responses. A late secondary neuronal response was significantly more likely in mice that were noise exposed over all other mature animals (Fig. 9*G*; noise-exposed: 7/31 vs non-noise-exposed: 4/77, *p* = 0.036, *N* = 23 animals, Fisher’s exact test for count data with Bonferroni correction for multiple comparisons), although delayed responses were present in some non-noise exposed mice (Fig. 8*C*, arrow). The observation that both NGAF and neuronal responses display secondary responses after noise exposure strengthens the correlation between NGAF and neuronal responses. However, it is unclear at this time if these are causally related, or independent responses to the initial damage.

Movie 3. Noise exposure causes delayed NGAF and neuronal responses in an adult *Th^2A-CreER^*;GCaMP6f mouse. Video created from images taken every 80 ms before and after a photoablation in an adult *Th^2A-CreER^*;GCaMP6f mouse 7 d after noise exposure. Video has been sped up 20 from raw footage. The word ablation appears in the top left corner of the video frame for 2 s after the photoablation event occurs. Several minutes after photoablation (here at the 20-s mark) neurons and epithelial cells show a delayed increase in fluorescence around the site of damage. 10.1523/ENEURO.0383-21.2021.video.3

## Discussion

### GCaMP6f expression in type II afferents reveals a calcium response to focal ablation in mature and noise exposed mice

Calcium imaging using Cre-driven GCaMP6f expression expands functional studies of type II afferents in the cochlea. This method coupled with two-photon microscopy of an otic capsule preparation enables recording of type II activity throughout the entire cochlea of mature mice. Calcium signals in type II afferent dendrites extend and add to the previous description of electrical signals evoked by ATP and OHC rupture in prehearing animals (Weisz et al., 2009, 2012, 2014; Liu et al., 2015). The fluorescent response to calcium immediately after focal laser ablation is similar in time course to the depolarization observed in prehearing type II afferents after OHC rupture (Liu et al., 2015). But in addition, these studies of the mature, and posttrauma cochlear tissue have revealed activity patterns not seen in younger cochleas. Development of more Cre mouse lines to target subtypes of type I or type II afferents specifically provides an opportunity to expand the use of this technique for cochlear neuroscience.

Focal ablation produced similar responses in type II afferents distinguished by gene expression and location along the cochlear tonotopic axis. The genes that define the two mouse models used in the majority of this study, *Th* and *Drd2*, are part of the dopamine production and response pathway and target apical and basal type II afferents, respectively (Beaulieu and Gainetdinov, 2011; Daubner et al., 2011; Vyas et al., 2017, 2019; Wu et al., 2018). The genes encoding CGRP-α and SERT also mark specific populations of type II afferents along the tonotopic axis (Vyas et al., 2019). Targeting these various neurotransmitter and neuropeptide pathways may help tease apart functional differences between these genetically distinct type II afferents.

Maturation and the onset of hearing had significant effects on type II afferent responses to damage. Mature type II afferents can respond to focal damage of the organ of Corti, albeit with lower probability and longer time course, when compared with prehearing neurons. The reduction in neuronal response probability correlated with a reduction in the frequency of supporting cell activity (here called NGAF activity) in mature tissue, implying that epithelial cell activity is related to type II afferent responses, presumably through the release of ATP or other activators (Cook and McCleskey, 2002; Gale et al., 2004; Tritsch et al., 2007; Talagas et al., 2020).

### ATP release underlies epithelial cell and type II afferent response to focal ablation in prehearing cochleas

ATP is important for refining neuronal circuitry and activity in prehearing animals for multiple cochlear cell types including type II afferents (Tritsch et al., 2007, 2010; Ceriani et al., 2019). Beyond spontaneous events, ATP release in the cochlea can be elicited by cell rupture and then propagated through ATP mediated ATP release in the cochlear epithelium (Cook and McCleskey, 2002; Gale et al., 2004). This amplification after damage is evident in the widespread NGAF and type II neuron responses. In prehearing animals, the proportion of type II afferents that experienced calcium transients to acute photoablation was reduced in the presence of PPADS, indicating that ATP release from epithelial cells increases the number of activated type II afferent neurons. This corresponds with previous observations that ATP-evoked inward currents of type II afferents were abbreviated similarly by PPADS or connexin block (Liu et al., 2015) as though produced by connexin-dependent release of ATP from supporting cells. Unfortunately, direct observation of calcium waves spreading throughout the epithelium was hindered by the seconds-long lag in recording following photoablation. Alternative approaches that reduce the lag time could clarify how the calcium wave propagates after insult and reveal faster calcium transients.

### NGAF is correlated with neuronal activity

In mature cochle as, the NGAF magnitude was smaller than in prehearing cochleas, and fewer type II afferents responded after damage. The remaining NGAF activity is no longer spontaneous and thus most likely represents the “slow waves” observed by Sirko and colleagues, where ATP-independent calcium responses spread among the supporting cells of the organ of Corti (Sirko et al., 2019). Additional experiments are necessary to learn if there is a causal link between epithelial cell activity and neuron activation especially in the mature cochlea. The location, timing, and ATP-dependence of NGAF activity suggest that it represents epithelial cell activity; however, these measurements were indirect. An ideal mouse model would combine the ability to compare NGAF activity with direct measurements of epithelial cell calcium signals while maintaining the ability to measure neuronal responses. Serendipitously, *Tac1^Cre^* expression of GCaMP6f varied along the cochlear length to provide that opportunity. In the mid-cochlea, *Tac1^Cre^*;GCaMP6f labeled all type II neurons, but produced a mosaic of epithelial expression, revealing the tight correlation between epithelial cell and type II afferent activity. Average NGAF activity and GCaMP6f responses had a similarly extended response duration after focal ablation, although individual epithelial cell GCaMP6f signals were markedly shorter in duration. The similarity of NGAF event and epithelial cell GCaMP6f activity supports the claim that NGAF activity reflects the calcium responses of the epithelial cells. Thus, the larger changes in NGAF activity with maturation reveal the shift from spontaneous, fast events to infrequent, slow waves after the onset of hearing.

### Similarities between type II afferents and somatic nociceptors

Building on previous studies of gene expression, morphology and function, the expression of *Tac1* strengthens the hypothesis that type II afferents are cochlear nociceptors. Type II afferents and somatosensory C-fibers both have a thin, unmyelinated, highly branched morphology. C-fibers in the somatosensory system report tissue damage (Cook and McCleskey, 2002); similarly, prehearing type II afferent neurons respond to cell rupture (Liu et al., 2015). C-fibers and type II afferents also share gene expression including *Tac1*, as well as *Calca* and *Th* (Le Pichon and Chesler, 2014; Wu et al., 2018). Somatosensory nociceptors can become sensitized and release CGRP-α and substance P after tissue damage (Andrew and Greenspan, 1999; Li et al., 2008; Smith et al., 2013; Murthy et al., 2018). Previous studies have shown an effect of substance P directly on SGNs and a protective effect of substance P after acoustic trauma (Nario et al., 1995; Ito et al., 2002; Sun et al., 2004; Kanagawa et al., 2014). Therefore, additional study of the effect of substance P on type II afferents in the context of acute damage is needed.

Calcium imaging offers an opportunity to study changes in mature type II activity following acoustic trauma. Delayed responses occurring minutes after the initial tissue damage in type II afferents and epithelial cells became more common after acoustic trauma. The timing of the onset of delayed responses suggests that these could occur via G-protein-coupled receptor pathways and/or posttranslational modifications, although the specific mechanism is unknown (Hoare et al., 2020; Gold, 1999). Increased somatosensory pain can occur within the same time frame (2–5 min) as the delayed cochlear responses to acute tissue damage after acoustic trauma. Increased somatic pain also involves inflammation leading to NMDA receptor subunit phosphorylation downstream of PKC (Guo et al., 2002). It remains to be seen whether inflammation plays a role in the prolonged response to acute damage in the cochlea.

Another possible mechanism of delayed responses can be seen in another somatosensory system. Spinal cord circuits involved in itch rely on burst action potential firing to induce neuropeptide release that drives sustained firing in downstream neurons by closing KCNQ channels (Pagani et al., 2019). KCNQ channels were implicated in the type II afferent response to damage (Liu et al., 2015) and type II afferents appear to express genes for KCNQ channels and several neuropeptides including CGRP and Substance P (Lallemend et al., 2003; Shrestha et al., 2018; Wu et al., 2018; Vyas et al., 2019) indicating that neuropeptide release in the noise-damaged cochlea could explain the sustained calcium activity observed in type II afferent dendrites following acoustic trauma.

### Putative role of type II afferents in pathologic hearing

Hearing loss subsequent to acoustic trauma is correlated with increased synaptic ribbons in the OHCs, predicting a functionally significant increase in the number of type II afferent action potentials (Wood et al., 2021). The prolonged depolarization of type II afferents indicated by GCaMP6f imaging could further lower the threshold for activation by transmission from OHCs during sound. If type II afferents are equivalent to nociceptors, their increased activation after hearing loss, in combination with synaptopathic loss of type I afferent activity (Stamataki et al., 2006; Kujawa and Liberman, 2009), could cause innocuous sound to become aversive. However, it is still unclear whether type II afferents contribute to pathologic changes following traumatic noise exposure. For example, painful hyperacusis is a common sequela of acoustic trauma and is characterized by a decrease in the intensity at which sound becomes painful, mirroring the greater sensitivity and longer duration responses to stimulation seen in somatic pain syndromes such as allodynia and hyperalgesia (Jensen and Finnerup, 2014; Pienkowski et al., 2014; Tyler et al., 2014). Modulation of type II activity therefore provides a possible therapeutic target for damage-induced hyperacusis. Further study is required however to develop a sufficiently robust animal model for hyperacusis to test the connection between hyperacusis and altered type II afferent function.

## Synthesis

Reviewing Editor: Christine Portfors, Washington State University Decisions are customarily a result of the Reviewing Editor and the peer reviewers coming together and discussing their recommendations until a consensus is reached. When revisions are invited, a fact-based synthesis statement explaining their decision and outlining what is needed to prepare a revision will be listed below. The following reviewer(s) agreed to reveal their identity: NONE. Note: If this manuscript was transferred from JNeurosci and a decision was made to accept the manuscript without peer review, a brief statement to this effect will instead be what is listed below.

Thank you for addressing the previous reviewers' comments so fully.

## References

[B201] Abraira VE, Kuehn ED, Chirila AM, Springel MW, Toliver AA, Zimmerman AL, Orefice LL, Boyle KA, Bai L, Song BJ, Bashista KA, O’Neill TG, Zhuo J, Tsan C, Hoynoski J, Rutlin M, Kus L, Niederkofler V, Watanabe M, Dymecki SM, Nelson SB, Heintz N, Hughes DI, Ginty DD (2017) The cellular and synaptic architecture of the mechanosensory dorsal horn. Cell 168(1–2):310.e19–310.e19. 10.1073/pnas.0800793105 28041852 PMC5236062

[B1] Andrew D, Greenspan JD (1999) Mechanical and heat sensitization of cutaneous nociceptors after peripheral inflammation in the rat. J Neurophysiol 82:2649–2265. 10.1152/jn.1999.82.5.2649 10561434

[B2] Anselmi F, Hernandez VH, Crispino G, Seydel A, Ortolano S, Roper SD, Kessaris N, Richardson W, Rickheit G, Filippov MA, Monyer H, Mammano F (2008) ATP release through connexin hemichannels and gap junction transfer of second messengers propagate Ca^2+^ signals across the inner ear. Proc Natl Acad Sci USA 105:18770–18775. 10.1073/pnas.0800793105 19047635 PMC2596208

[B3] Babola TA, Kersbergen CJ, Wang HC, Bergles DE (2020) Purinergic Signaling in cochlear supporting cells reduces hair cell excitability by increasing the extracellular space. Elife 9:e52160. 10.7554/eLife.52160 31913121 PMC7015667

[B4] Beaulieu JM, Gainetdinov RR (2011) The physiology, signaling, and pharmacology of dopamine receptors. Pharmacol Rev 63:182–217. 10.1124/pr.110.002642 21303898

[B5] Berglund AM, Ryugo DK (1987) Hair cell innervation by spiral ganglion neurons in the mouse. J Comp Neurol 255:560–570. 10.1002/cne.902550408 3819031

[B6] Brown MC (1994) Antidromic responses of single units from the spiral ganglion. J Neurophysiol 71:1835–1847. 10.1152/jn.1994.71.5.1835 8064351

[B7] Ceriani F, Hendry A, Jeng JY, Johnson SL, Stephani F, Olt J, Holley MC, Mammano F, Engel J, Kros CJ, Simmons DD, Marcotti W (2019) Coordinated calcium signalling in cochlear sensory and non‐sensory cells refines afferent innervation of outer hair cells. EMBO J 38:e99839. 10.15252/embj.201899839 30804003 PMC6484507

[B8] Cook SP, McCleskey EW (2002) Cell damage excites nociceptors through release of cytosolic ATP. Pain 95:41–47. 10.1016/s0304-3959(01)00372-4 11790466

[B9] Daubner SC, Le T, Wang S (2011) Tyrosine hydroxylase and regulation of dopamine synthesis. Arch Biochem Biophys 508:1–12. 10.1016/j.abb.2010.12.017 21176768 PMC3065393

[B10] Flores EN, Duggan A, Madathany T, Hogan AK, Márquez FG, Kumar G, Seal RP, Edwards RH, Liberman MC, García-Añoveros J (2015) A non-canonical pathway from cochlea to brain signals tissue-damaging noise. Curr Biol 25:606–612. 10.1016/j.cub.2015.01.009 25639244 PMC4348215

[B11] Gale JE, Piazza V, Ciubotaru CD, Mammano F (2004) A mechanism for sensing noise damage in the inner ear. Curr Biol 14:526–529. 10.1016/j.cub.2004.03.002 15043820

[B12] Ghimire SR, Deans MR (2019) Frizzled3 and Frizzled6 cooperate with Vangl2 to direct cochlear innervation by type II spiral ganglion neurons. J Neurosci 39:8013–8023. 10.1523/JNEUROSCI.1740-19.2019 31462532 PMC6786817

[B120] Gold MS (1999) Tetrodotoxin-resistant Na+ currents and inflammatory hyperalgesia. Proceedings of the National Academy of Sciences 96:7645–7649. 10.1073/pnas.96.14.7645 PMC33595 10393874

[B13] Guo W, Zou S, Guan Y, Ikeda T, Tal M, Dubner R, Ren K (2002) Tyrosine phosphorylation of the NR2B subunit of the NMDA receptor in the spinal cord during the development and maintenance of inflammatory hyperalgesia. J Neurosci 22:6208–6217. 12122079 10.1523/JNEUROSCI.22-14-06208.2002 PMC6757905

[B14] Hoare SRJ, Tewson PH, Quinn AM, Hughes TE, Bridge LJ (2020) Analyzing kinetic signaling data for G-protein-coupled receptors. Sci Rep 10:12263. 10.1038/s41598-020-67844-3 32704081 PMC7378232

[B15] Ito K, Rome C, Bouleau Y, Dulon D (2002) Substance P mobilizes intracellular calcium and activates a nonselective cation conductance in rat spiral ganglion neurons. Eur J Neurosci 16:2095–2102. 10.1046/j.1460-9568.2002.02292.x 12473077

[B16] Jensen TS, Finnerup NB (2014) Allodynia and hyperalgesia in neuropathic pain: clinical manifestations and mechanisms. Lancet Neurol 13:924–935. 10.1016/S1474-4422(14)70102-4 25142459

[B17] Johnson KR, Erway LC, Cook SA, Willott JF, Zheng QY (1997) A major gene affecting age-related hearing loss in C57BL/6J mice. Hear Res 114:83–92. 10.1016/S0378-5955(97)00155-X 9447922

[B18] Johnson KR, Tian C, Gagnon LH, Jiang H, Ding D, Salvi R (2017) Effects of Cdh23 single nucleotide substitutions on age-related hearing loss in C57BL/6 and 129S1/Sv mice and comparisons with congenic strains. Sci Rep 7:44450. 10.1038/srep44450 28287619 PMC5347380

[B19] Kanagawa E, Sugahara K, Hirose Y, Mikuriya T, Shimogori H, Yamashita H (2014) Effects of substance P during the recovery of hearing function after noise-induced hearing loss. Brain Res 1582:187–196. 10.1016/j.brainres.2014.07.024 25064433

[B20] Kebabian JW, Calne DB (1979) Multiple receptors for dopamine. Nature 277:93–96. 10.1038/277093a0 215920

[B21] Kestell GR, Anderson RL, Clarke JN, Haberberger RV, Gibbins IL (2015) Primary afferent neurons containing calcitonin gene-related peptide but not substance P in forepaw skin, dorsal root ganglia, and spinal cord of mice. J Comp Neurol 523:2555–2569. 10.1002/cne.23804 26010480

[B22] Kiang NY, Rho JM, Northrop CC, Liberman MC, Ryugo DK (1982) Hair-cell innervation by spiral ganglion cells in adult cats. Science 217:175–177. 10.1126/science.7089553 7089553

[B23] Krause JE, Chirgwin M, Carter MS, Xu ZS, Hershey AD (1987) Three rat preprotachykinin mRNAs encode the neuropeptides substance P and neurokinin A. Proc Natl Acad Sci USA 84:881–885. 10.1073/pnas.84.3.881 2433692 PMC304320

[B24] Kujawa SG, Liberman MC (2009) Adding insult to injury: cochlear nerve degeneration after “temporary” noise-induced hearing loss. J Neurosci 29:14077–14085. 10.1523/JNEUROSCI.2845-09.2009 19906956 PMC2812055

[B25] Lahne M, Gale JE (2008) Damage-induced activation of ERK1/2 in cochlear supporting cells is a hair cell death-promoting signal that depends on extracellular ATP and calcium. J Neurosci 28:4918–4928. 10.1523/JNEUROSCI.4914-07.2008 18463245 PMC6670733

[B26] Lahne M, Gale JE (2010) Damage-induced cell-cell communication in different cochlear cell types via two distinct ATP-dependent Ca waves. Purinergic Signal 6:189–200. 10.1007/s11302-010-9193-8 20806011 PMC2912991

[B27] Lallemend F, Lefebvre PP, Hans G, Rigo JM, Van de Water TR, Moonen G, Malgrange B (2003) Substance P protects spiral ganglion neurons from apoptosis via PKC-Ca^2+^-MAPK/ERK pathways. J Neurochem 87:508–521. 10.1046/j.1471-4159.2003.02014.x 14511128

[B28] Lauer AM, May BJ (2011) The medial olivocochlear system attenuates the developmental impact of early noise exposure. J Assoc Res Otolaryngol 12:329–343. 10.1007/s10162-011-0262-7 21347798 PMC3085693

[B29] Le Pichon CE, Chesler AT (2014) The functional and anatomical dissection of somatosensory subpopulations using mouse genetics. Front Neuroanat 8:21. 10.3389/fnana.2014.00021 24795573 PMC4001001

[B30] Li D, Ren Y, Xu X, Zou X, Fang L, Lin Q (2008) Sensitization of primary afferent nociceptors induced by intradermal capsaicin involves the peripheral release of calcitonin gene-related peptide driven by dorsal root reflexes. J Pain 9:1155–1168. 10.1016/j.jpain.2008.06.011 18701354 PMC2642671

[B31] Lina IA, Lauer AM (2013) Rapid measurement of auditory filter shape in mice using the auditory brainstem response and notched noise. Hear Res 298:73–79. 10.1016/j.heares.2013.01.002 23347916 PMC3639490

[B32] Liberman MC (1982) The cochlear frequency map for the cat: labeling auditory-nerve fibers of known characteristic frequency. J Acoust Soc Am 72:1441–1449. 10.1121/1.388677 7175031

[B33] Liu C, Glowatzki E, Fuchs PA (2015) Unmyelinated type II afferent neurons report cochlear damage. Proc Natl Acad Sci USA 112:14723–14727. 10.1073/pnas.1515228112 26553995 PMC4664349

[B34] Martinez-Monedero R, Liu C, Weisz C, Vyas P, Fuchs PA, Glowatzki E (2016) GluA2-containing AMPA receptors distinguish ribbon-associated from ribbonless afferent contacts on rat cochlear hair cells. eNeuro 3:ENEURO.0078-16.2016. 10.1523/ENEURO.0078-16.2016 PMC4874539 27257620

[B35] Molinoff PB, Axelrod J (1971) Biochemistry of catecholamines. Annu Rev Biochem 40:465–500. 10.1146/annurev.bi.40.070171.002341 4399447

[B36] Murthy SE, Loud MC, Daou I, Marshall KL, Schwaller F, Kühnemund J, Francisco AG, Keenan WT, Dubin AE, Lewin GR, Patapoutian A (2018) The mechanosensitive ion channel Piezo2 mediates sensitivity to mechanical pain in mice. Sci Transl Med 10:eaat9897. 10.1126/scitranslmed.aat9897 30305457 PMC6709986

[B37] Nario K, Kitano I, Mori N, Matsunaga T (1995) The action of substance P methyl ester on cochlear potentials in the guinea pig. Eur Arch Otorhinolaryngol 252:42–47. 10.1007/BF00171439 7536424

[B38] Pagani M, Albisetti GW, Sivakumar N, Wildner H, Santello M, Johannssen HC, Zeilhofer HU (2019) How gastrin-releasing peptide opens the spinal gate for itch. Neuron 103:102–117. 10.1016/j.neuron.2019.04.022 31103358 PMC6616317

[B39] Perkins RE, Morest DK (1975) A study of cochlear innervation patterns in cats and rats with the Golgi method and Nomarkski optics. J Comp Neurol 163:129–158. 10.1002/cne.901630202 1100684

[B40] Pienkowski M, Tyler RS, Roncancio ER, Jun HJ, Brozoski T, Dauman N, Coelho CB, Andersson G, Keiner AJ, Cacace AT, Martin N, Moore BCJ (2014) A review of hyperacusis and future direction: part II. Measurement, mechanisms, and treatment. Am J Audiol 23:420–436. 10.1044/2014_AJA-13-0037 25478787

[B41] Robertson D (1984) Horseradish peroxidase injection of physiologically characterized afferent and efferent neurones in the guinea pig spiral ganglion. Hear Res 15:113–121. 10.1016/0378-5955(84)90042-X 6490538

[B42] Robertson D, Sellick PM, Patuzzi R (1999) The continuing search for outer hair cell afferents in the guinea pig spiral ganglion. Hear Res 136:151–158. 10.1016/s0378-5955(99)00120-3 10511634

[B43] Shrestha BR, Chia C, Wu L, Kujawa SG, Liberman MC, Goodrich LV (2018) Sensory neuron diversity in the inner ear is shaped by activity. Cell 174:1229–1246. 10.1016/j.cell.2018.07.007 30078709 PMC6150604

[B44] Sirko P, Gale JE, Ashmore JF (2019) Intercellular Ca^2+^signalling in the adult mouse cochlea. J Physiol 597:303– 317. 10.1113/JP276400 30318615 PMC6312409

[B45] Sleigh JN, Weir GA, Schiavo G (2016) A simple, step-by-step dissection protocol for the rapid isolation of mouse dorsal root ganglia. BMC Res Notes 9:82. 10.1186/s13104-016-1915-8 26864470 PMC4750296

[B46] Smith AK, O’Hara CL, Stucky CL (2013) Mechanical sensitization of cutaneous sensory fibers in the spared nerve injury mouse model. Mol Pain 9:61. 10.1186/1744-8069-9-61 24286165 PMC3906996

[B47] Smith CA (1975) Innervation of the cochlea of the guinea pig by use of the Golgi stain. Ann Otol Rhinol Laryngol 84:443–458. 10.1177/000348947508400403 50759

[B48] Stamataki S, Francis HW, Lehar M, May BJ, Ryugo DK (2006) Synaptic alterations at inner hair cells precede spiral ganglion cell loss in aging C57BL/6J mice. Hear Res 221:104–118. 10.1016/j.heares.2006.07.014 17005343

[B49] Sun W, Ding DL, Wang P, Sun J, Jin X, Salvi RJ (2004) Substance P inhibits potassium and calcium currents in inner ear spiral ganglion neurons. Brain Res 1012:82–92. 10.1016/j.brainres.2004.03.051 15158164

[B50] Talagas M, Lebonvallet N, Berthod F, Misery L (2020) Lifting the veil on the keratinocyte contribution to cutaneous nociception. Protein Cell 11:239–250. 10.1007/s13238-019-00683-9 31907794 PMC7093357

[B51] Tritsch NX, Eunyoung Y, Gale JE, Glowatzki E, Bergles DE (2007) The origin of spontaneous activity in the developing auditory system. Nature 450:50–55. 10.1038/nature06233 17972875

[B52] Tritsch NX, Zhang Y, Ellis-Davies G, Bergles DE (2010) ATP-induced morphological changes in supporting cells of the developing cochlea. Purinergic Signal 6:155–166. 10.1007/s11302-010-9189-4 20806009 PMC2912990

[B53] Tyler RS, Pienkowski M, Roncancio ER, Jun HJ, Brozoski T, Dauman N, Dauman N, Andersson G, Keiner AJ, Cacace AT, Martin N, Moore BCJ (2014) A review of hyperacusis and future direction: part I. Definitions and manifestations. Am J Audiol 23:402–419. 10.1044/2014_AJA-14-0010 25104073

[B54] Vyas P, Wu JS, Zimmerman A, Fuchs PA, Glowatzki E (2017) Tyrosine hydroxylase expression in type II cochlear afferents in mice. J Assoc Res Otolaryngol 18:139–151. 10.1007/s10162-016-0591-7 27696081 PMC5243262

[B55] Vyas P, Wu JS, Jimenez A, Glowatzki E, Fuchs PA (2019) Characterization of transgenic mouse lines for labeling type I and type II afferent neurons in the cochlea. Sci Rep 9:5549. 10.1038/s41598-019-41770-5 30944354 PMC6447598

[B56] Weissner W, Winterson BJ, Stuart-Tilley A, Devor M, Bove GM (2006) Time course of substance P expression in dorsal root ganglia following complete spinal nerve transection. J Comp Neurol 497:78–87. 10.1002/cne.20981 16680762 PMC2571959

[B57] Weisz CJC, Glowatzki E, Fuchs PA (2009) The postsynaptic function of type II cochlear afferents. Nature 461:1126–1129. 10.1038/nature08487 19847265 PMC2785502

[B58] Weisz CJC, Lehar M, Hiel H, Glowatzki E, Fuchs PA (2012) Synaptic transfer from outer hair cells to type II afferent fibers in the rat cochlea. J Neurosci 32:9528–9536. 10.1523/JNEUROSCI.6194-11.2012 22787038 PMC3433252

[B59] Weisz CJC, Glowatzki E, Fuchs PA (2014) Excitability of type II cochlear afferents. J Neurosci 34:2365–2373. 10.1523/JNEUROSCI.3428-13.2014 24501375 PMC3913877

[B60] Weisz CJC, Williams SPG, Eckard CS, Divito CB, Ferreira DW, Fantetti KN, Dettwyler SA, Cai HM, Rubio ME, Kandler K, Seal RP (2021) Outer hair cell glutamate signaling through type II spiral ganglion afferents activates neurons in the cochlear nucleus in response to nondamaging sounds. J Neurosci 41:2930–2943. 10.1523/JNEUROSCI.0619-20.2021 33574178 PMC8018895

[B61] Wood MB, Nowak N, Mull K, Goldring A, Lehar M, Fuchs PA (2021) Acoustic trauma increases ribbon number and size in outer hair cells of the mouse cochlea. J Assoc Res Otolaryngol 22:19–31. 10.1007/s10162-020-00777-w 33151428 PMC7822997

[B62] Wu JS, Vyas P, Glowatzki E, Fuchs PA (2018) Opposing expression gradients of calcitonin-related polypeptide alpha (Calca/Cgrpα) and tyrosine hydroxylase (Th) in type II afferent neurons of the mouse cochlea. J Comp Neurol 526:425–438. 10.1002/cne.24341 29055051 PMC5975645

[B63] Wu JS, Yi E, Manca M, Javaid H, Lauer AM, Glowatzki E (2020) Sound exposure dynamically induces dopamine synthesis in cholinergic LOC efferents for feedback to auditory nerve fibers. Elife 9:e52419. 10.7554/eLife.52419 31975688 PMC7043886

